# ADHD drug treatment and risk of suicidal behaviours, substance misuse, accidental injuries, transport accidents, and criminality: emulation of target trials

**DOI:** 10.1136/bmj-2024-083658

**Published:** 2025-08-13

**Authors:** Le Zhang, Nanbo Zhu, Arvid Sjölander, Mikail Nourredine, Lin Li, Miguel Garcia-Argibay, Ralf Kuja-Halkola, Isabell Brikell, Paul Lichtenstein, Brian M D’Onofrio, Henrik Larsson, Samuele Cortese, Zheng Chang

**Affiliations:** 1 Department of Medical Epidemiology and Biostatistics, Karolinska Institutet, Stockholm, Sweden; 2 Division of Clinical Geriatrics, Department of Neurobiology, Care Sciences and Society, Karolinska Institutet, Stockholm, Sweden; 3 Centre for Innovation in Mental Health, School of Psychology, Faculty of Environmental and Life Sciences, and Clinical and Experimental Sciences (CNS and Psychiatry), Faculty of Medicine, University of Southampton, Southampton, UK; 4 Department of Biostatistics-Bioinformatics, Hospices Civils de Lyon, Lyon, France; 5 Université Claude Bernard Lyon 1, LBBE, UMR 5558, CNRS, VAS, Villeurbanne, France; 6 School of Medical Sciences, Faculty of Medicine and Health, Örebro University, Örebro, Sweden; 7 Centre for Population Health, Research Department, Division for Mental Health, Haukeland University Hospital, Bergen, Norway; 8 Department of Global Public Health and Primary Care, University of Bergen, Bergen, Norway; 9 Department of Biomedicine, Aarhus University, Aarhus C, Denmark; 10 Department of Psychological and Brain Sciences, Indiana University, Bloomington, IN, USA; 11 Clinical and Experimental Sciences (CNS and Psychiatry), Faculty of Medicine, University of Southampton, Southampton, UK; 12 Hampshire and Isle of Wight Healthcare NHS Foundation Trust, Southampton, UK; 13 Hassenfeld Children’s Hospital at NYU Langone, New York University Child Study Center, New York City, NY, USA; 14 DiMePRe-J-Department of Precision and Regenerative Medicine-Jonic Area, University of Bari “Aldo Moro,” Bari, Italy

## Abstract

**Objective:**

To examine the effects of drug treatment for attention deficit/hyperactivity disorder (ADHD) on suicidal behaviours, substance misuse, accidental injuries, transport accidents, and criminality.

**Design:**

Emulation of target trials.

**Setting:**

Linkage of national registers in Sweden, 2007-20.

**Participants:**

People aged 6-64 years with a new diagnosis of ADHD, who either started or did not start drug treatment for ADHD within three months of diagnosis.

**Main outcome measures:**

First and recurrent events of five outcomes over two years after ADHD diagnosis: suicidal behaviours, substance misuse, accidental injuries, transport accidents, and criminality.

**Results:**

Of 148 581 individuals with ADHD (median age 17.4 years; 41.3% female), 84 282 (56.7%) started drug treatment for ADHD, with methylphenidate being the most commonly prescribed at initiation (74 515; 88.4%). Drug treatment for ADHD was associated with reduced rates of the first occurrence of suicidal behaviours (weighted incidence rates 14.5 per 1000 person years in the initiation group versus 16.9 in the non-initiation group; adjusted incidence rate ratio 0.83, 95% confidence interval 0.78 to 0.88), substance misuse (58.7 *v* 69.1 per 1000 person years; 0.85, 0.83 to 0.87), transport accidents (24.0 *v* 27.5 per 1000 person years; 0.88, 0.82 to 0.94), and criminality (65.1 *v* 76.1 per 1000 person years; 0.87, 0.83 to 0.90), whereas the reduction was not statistically significant for accidental injuries (88.5 *v* 90.1 per 1000 person years; incidence rate ratio 0.98, 0.96 to 1.01). The reduced rates were more pronounced among individuals with previous events, with incidence rate ratios ranging from 0.79 (0.72 to 0.86) for suicidal behaviours to 0.97 (0.93 to 1.00) for accidental injuries. For recurrent events, drug treatment for ADHD was significantly associated with reduced rates of all five outcomes, with incidence rate ratios of 0.85 (0.77 to 0.93) for suicidal behaviours, 0.75 (0.72 to 0.78) for substance misuse, 0.96 (0.92 to 0.99) for accidental injuries, 0.84 (0.76 to 0.91) for transport accidents, and 0.75 (0.71 to 0.79) for criminality.

**Conclusions:**

Drug treatment for ADHD was associated with beneficial effects in reducing the risks of suicidal behaviours, substance misuse, transport accidents, and criminality but not accidental injuries when considering first event rate. The risk reductions were more pronounced for recurrent events, with reduced rates for all five outcomes. This target trial emulation study using national register data provides evidence that is representative of patients in routine clinical settings.

## Introduction

Attention deficit/hyperactivity disorder (ADHD) is a prevalent neurodevelopmental disorder, affecting approximately 5% of children and 2.5% of adults worldwide.^1^ ^2^ ^3^ Although typically diagnosed in childhood, its impairing symptoms often persist into adulthood.^4^ Beyond core symptoms, ADHD is linked to a range of adverse functional outcomes, including increased risks of suicidal behaviours, substance misuse, accidental injuries, transport accidents, and criminality.^1^ ^5^ Treatment for ADHD includes drug, non-drug, and combined approaches. Although non-drug treatment is often recommended for younger children or milder cases, drug treatment (including stimulants and non-stimulants) is commonly used in the management of school aged and older individuals with ADHD. Prescriptions of drugs for ADHD have risen markedly in recent years worldwide, sparking intense debate on their effectiveness and safety.^6^ ^7^

Randomised controlled trials have shown the beneficial effects of drug treatment for ADHD in alleviating core symptoms.^8^ However, evidence from randomised controlled trials remains limited or inconclusive for broader and important clinical outcomes such as suicidal behaviours and substance use disorder.^9^ ^10^ ^11^ ^12^ Moreover, randomised controlled trials often exclude a substantial population of patients seen in clinical practice—around half of those receiving drugs for ADHD,^13^ thereby limiting the generalisability to the entire ADHD population. In this context, pharmacoepidemiological studies using routinely collected data offer opportunities to assess the benefits and risks of ADHD drug treatment on broader outcomes.^14^ ^15^ In particular, studies using within individual designs have linked use of drugs for ADHD to reduced risks of suicidal behaviours,^16^ ^17^ ^18^ substance misuse,^19^ ^20^ accidental injuries,^21^ transport accidents,^22^ ^23^ and criminality.^24^ Although effectively controlled for time invariant confounders, these studies remain susceptible to time varying confounding and carryover effects,^25^ and their reliance on treated patients who have experienced the outcomes of interest limits both the generalisability and comparability to trial findings. Thus, rigorous population based studies using routine clinical data, designed to ensure representativeness and comparability to trials, are needed.

To overcome these limitations, this study for the first time applied the target trial emulation framework to examine the effects of drug treatment for ADHD on five critical outcomes—suicidal behaviours, substance misuse, accidental injuries, transport accidents, and criminality. This approach enhances causal inference by mimicking the design principles of a randomised controlled trial within an observational context and provides estimates of treatment effects for the entire ADHD population from routine practice. Leveraging Swedish national registers, we examined both first and recurrent events, reflecting the recurrent nature of these outcomes. The selection of outcomes was made in consultation with people with lived experience, aligning with the practical needs of those affected by ADHD.

## Methods

### Data sources

We obtained data by linking multiple Swedish registers using the unique personal identification number assigned to every resident in Sweden.^26^ The Swedish Total Population Register covers demographic information on all Swedish inhabitants since 1968.^27^ It also contains information on all migrations in or out of Sweden. The National Patient Register includes data on inpatient care since 1973 and outpatient care since 2001,^28^ based on the International Classification of Diseases (ICD) in its eighth (ICD-8; 1969-86), ninth (ICD-9; 1987-96), and tenth (ICD-10; since 1997) revisions. The Prescribed Drug Register includes detailed information on all dispensed drugs in Sweden since 1 July 2005, based on the Anatomical Therapeutic Chemical (ATC) classification.^29^ The Cause of Death Register contains information on all registered deaths since 1952,^30^ including underlying and contributing causes of death. The National Crime Register provides information on convicted crime since 1973.^31^ The Longitudinal Integration Database for Health Insurance and Labor Studies integrates data from the labour market, and educational and social sectors, covering the entire Swedish population aged 16 or older since 1990.^32^

### Study design and study cohort

**Fig 1:**
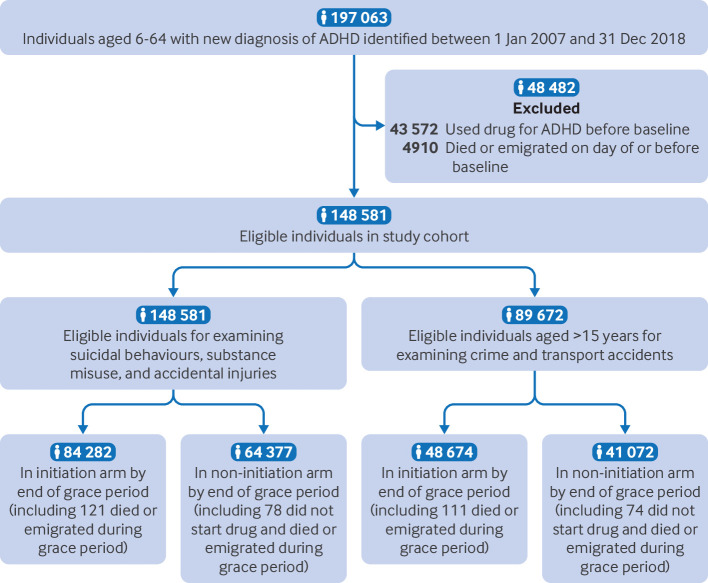
Selection of study population. ADHD=attention deficit/hyperactivity disorder

We applied the target trial emulation framework to estimate the effects of drug treatment for ADHD on five outcomes (see supplementary table A for the protocol of the target trials). We identified all Swedish residents aged 6-64 years who had an incident diagnosis of ADHD (ICD-10 code: F90) between 1 January 2007 and 31 December 2018. In Sweden, people referred or seeking care for ADHD undergo a thorough neuropsychiatric assessment at specialist psychiatric services, using diagnostic criteria in line with the Diagnostic and Statistical Manual of Mental Disorders.^33^ ^34^ To exclude prevalent users, we included only individuals who had no drug treatment for ADHD dispensed for at least 18 months before their ADHD diagnosis.^35^ We did analyses of criminality and transport accidents in a sub-cohort aged 15-64 years, as the minimum legal age for criminal responsibility and driving in Sweden is 15 (fig 1).

We compared two treatment strategies: starting drug treatment for ADHD within three months after diagnosis and remaining on the prescribed drug versus not starting drug treatment for ADHD during the follow-up. We focused on the effect of sustained treatment—that is, the observational analogue of “per protocol” effects (detailed in the statistical analysis), up to two years of follow-up. We a priori chose a per protocol analysis, given that treatment discontinuation is common with ADHD drug treatment and that a true intention-to-treat effect cannot be fully emulated without randomisation.^36^ ^37^ Drugs licensed for ADHD treatment in Sweden during the study period included amphetamine (ATC code: N06BA01), atomoxetine (N06BA09), dexamphetamine (N06BA02), guanfacine (C02AC02), lisdexamfetamine (N06BA12), and methylphenidate (N06BA04).

This study was pre-registered in the Open Science Framework (https://osf.io/y7fhj/) and is reported in line with the REporting of studies Conducted using Observational Routinely collected health Data-PharmacoEpidemiological research (RECORD-PE) guidelines.^38^

### Outcomes and follow-up

We included five outcomes: suicidal behaviours (ICD-10 codes X60-X84, Y10-Y34), substance misuse (F10-F19, T36-T51, X40-X49), accidental injuries (V, W, X00-X59), transport accidents (V01-V99), and criminality (any crime conviction). We identified these outcomes from the National Patient Register, the Cause of Death Register, and the National Crime Register (see supplementary table B for details). The National Patient Register has shown good diagnostic accuracy, with a median positive predictive value of 84% (interquartile range 72-93%)^39^; the Cause of Death Register captures more than 99% of deaths^40^; and the Swedish Crime Register has near complete national coverage, with convictions reflecting adjudicated cases owing to the absence of plea bargaining.^40^ ^41^ Follow-up began at the time of ADHD diagnosis—that is, time zero—and continued until the outcome of interest, death, emigration, two years after baseline, or 31 December 2020, whichever came first.

### Covariates

We included pre-specified covariates to control for potential confounding factors, guided by existing knowledge, previous studies,^42^ ^43^ and a directed acyclic graph (supplementary figure A). Baseline covariates included demographics (age at ADHD diagnosis, calendar year, sex, country of birth, and highest education level (primary or lower secondary/upper secondary/post-secondary or postgraduate/unknown, using parents’ education level for those younger than 25 years), psychiatric diagnoses (anxiety disorder, autism spectrum disorder, bipolar disorder, conduct disorder, depressive disorder, eating disorder, intellectual disability, personality disorder, schizophrenia, alcohol use disorder, and substance use disorders), physical conditions (cardiovascular diseases, epilepsy, type 2 diabetes, and hyperlipidaemia), history of the outcome event (suicidal behaviours, substance misuse, accidental injuries, transport accidents, or criminality), dispensations of other psychotropic drugs (antipsychotics, anxiolytics, hypnotics, and sedatives, antidepressants, antiepileptic drugs, anti-addiction drugs, and opioids), and health care use (number of outpatient visits and hospital admissions for psychiatric and non-psychiatric reasons) (table 1). We also defined time varying covariates from the previous month including the aforementioned diagnoses, dispensations, and healthcare use. These potential confounders were defined according to ICD and ATC codes (supplementary table C).

**Table 1:** Characteristics of study cohort at baseline. Values are numbers (percentages) unless stated otherwise

Characteristics	Overall* (n=148 581)	Initiation† (n=84 282)	Non-initiation† (n=64 377)
Median (IQR) age at baseline	17.4 (11.6-29.1)	16.4 (11.5- 27.8)	19.1 (11.9-30.6)]
Sex:			
Male	87 225 (58.7)	49 649 (58.9)	37 631 (58.5)
Female	61 356 (41.3)	34 633 (41.1)	26 746 (41.5)
Median (IQR) calendar year at baseline	2014 (2011-2016)	2014 (2011-2016)	2014 (2011-2016)
Country of birth:			
Sweden	136 947 (92.2)	78 195 (92.8)	58 817 (91.4)
Other‡	11 634 (7.8)	6087 (7.2)	5560 (8.6)
Education level at baseline§:			
Primary or lower secondary	24 784 (16.7)	12 774 (15.2)	12 023 (18.7)
Upper secondary	75 256 (50.6)	42 991 (51.0)	32 299 (50.2)
Post-secondary or postgraduate	47 340 (31.9)	27 945 (33.2)	19 411 (30.2)
Unknown	1201 (0.8)	572 (0.7)	644 (1.0)
Comorbidities at baseline:			
Anxiety disorders	15 094 (10.2)	7415 (8.8)	7691 (11.9)
Autism spectrum disorder	16 780 (11.3)	7856 (9.3)	8930 (13.9)
Bipolar disorder	6551 (4.4)	2973 (3.5)	3582 (5.6)
Conduct disorder	4806 (3.2)	2966 (3.5)	1840 (2.9)
Depressive disorder	32 147 (21.6)	16 695 (19.8)	15 481 (24.0)
Eating disorder	3575 (2.4)	1842 (2.2)	1734 (2.7)
Intellectual disability	3849 (2.6)	1637 (1.9)	2215 (3.4)
Personality disorder	8835 (5.9)	3981 (4.7)	4867 (7.6)
Schizophrenia	2789 (1.9)	1102 (1.3)	1699 (2.6)
Epilepsy	3215 (2.2)	1344 (1.6)	1875 (2.9)
Alcohol use disorder	12 991 (8.7)	6362 (7.5)	6663 (10.3)
Substance use disorder	13 951 (9.4)	6585 (7.8)	7407 (11.5)
Cardiovascular disease	5204 (3.5)	2300 (2.7)	2918 (4.5)
Type 2 diabetes	1135 (0.8)	469 (0.6)	669 (1.0)
Dyslipidaemia	586 (0.4)	258 (0.3)	330 (0.5)
Psychotropic drug use at baseline:			
Opioids¶	30 785 (20.7)	17 064 (20.2)	13 754 (21.4)
Antiepileptic drugs	11 255 (7.6)	5366 (6.4)	5899 (9.2)
Antipsychotics	15 561 (10.5)	7767 (9.2)	7823 (12.2)
Anxiolytics, hypnotics, and sedatives	60 212 (40.5)	33 297 (39.5)	26 964 (41.9)
Antidepressants	52 967 (35.6)	28 157 (33.4)	24 860 (38.6)
Anti-addiction drugs**	7673 (5.2)	3955 (4.7)	3740 (5.8)
No of previous hospital admissions for psychiatric reasons:			
0	124 250 (83.6)	72 331 (85.8)	51 948 (80.7)
1-2	15 544 (10.5)	7924 (9.4)	7642 (11.9)
3-4	3697 (2.5)	1769 (2.1)	1939 (3.0)
≥5	5090 (3.4)	2258 (2.7)	2848 (4.4)
No of previous outpatient visits for psychiatric reasons:			
0	80 026 (53.9)	47 341 (56.2)	32 705 (50.8)
1-4	39 693 (26.7)	22 062 (26.2)	17 657 (27.4)
5-9	13 969 (9.4)	7328 (8.7)	6654 (10.3)
≥10	14 893 (10.0)	7551 (9.0)	7361 (11.4)
No of previous hospital admissions for non-psychiatric reasons:			
0	79 251 (53.3)	46 449 (55.1)	32 826 (51.0)
1-2	48 599 (32.7)	27 450 (32.6)	21 174 (32.9)
3-4	11 608 (7.8)	6130 (7.3)	5488 (8.5)
≥5	9123 (6.1)	4253 (5.0)	4889 (7.6)
No of previous outpatient visits for non-psychiatric reasons:			
0	25 629 (17.2)	14 317 (17.0)	11 329 (17.6)
1-4	65 004 (43.7)	37 541 (44.5)	27 491 (42.7)
5-9	31 808 (21.4)	18 223 (21.6)	13 599 (21.1)
≥10	26 140 (17.6)	14 201 (16.8)	11 958 (18.6)

IQR=interquartile range.

* Assessed at baseline.

† Assessed at baseline. Patients who died or emigrated and did not start drug treatment for attention deficit/hyperactivity disorder during grace period (n=78) contributed to both treatment strategies.

‡ Including all countries other than Sweden.

§ For patients younger than 25 years, education level was replaced by parents’ highest education level.

¶ Refers to prescribed opioids in Prescription Drug Register.

** Including drugs used in nicotine dependence, drugs used in alcohol dependence, and drugs used in opioid dependence.

### Statistical analysis

The two treatment strategies considered in our main analysis were starting drug treatment for ADHD within three months of diagnosis and remaining on the prescribed therapy (initiation group) versus not starting drug treatment for ADHD during the follow-up (non-initiation group). To estimate the average treatment effect of sustained ADHD drug treatment on five outcomes over the two year period for the entire study population, we applied a three step approach—cloning, censoring, and inverse probability weighting—designed to emulate the key features of randomised controlled trials and eliminate immortal time bias (supplementary figure B).^44^ ^45^ Firstly, in the cloning step, we created a dataset with two identical copies (clones) of each eligible individual at baseline. One clone was assigned to the treatment strategy of starting ADHD drug treatment within three months of diagnosis and remaining on treatment, and the other one was assigned to the strategy of not starting ADHD drug treatment during the follow-up. This step ensured alignment of treatment assignment with the start of follow-up and eliminated baseline confounding.^45^ ^46^ Secondly, in the censoring step, we assessed whether each clone adhered to the assigned treatment strategy at monthly intervals and censored them when they deviated from the assigned treatment strategy. Clones in the initiation group were censored if they had not started treatment by the end of grace period or discontinued/switched drug treatment after the grace period. Clones in the non-initiation group were censored on receipt of any ADHD drug treatment. Thirdly, in the weighting step, we applied pooled logistic regression models to calculate time varying inverse probability of censoring weights. These models included time and all time fixed and time varying covariates described above, to account for potential selection bias induced by the artificial censoring in the second step.^47^ Weights were truncated at the 99.5th centile to reduce the influence of extreme values (see supplementary methods for details).

To assess covariate balance at the end of the grace period (three months after ADHD diagnosis), we calculated standardised mean differences, with a difference <0.10 indicating sufficient balance.^48^ We fitted separate models for the five outcomes of interest by using weighted pooled logistic regression, regressing the outcome on treatment and time, which approximates the incidence rate ratio.^49^ We applied non-parametric bootstrapping with 500 full re-samples of individuals from the cohort to calculate the 95% confidence intervals.

In secondary analyses, we examined the association between drug treatment for ADHD and recurrent events of the five outcomes. To minimise misclassification of recurring treatment visits as outcome events, we allowed a maximum of one event a month. In these recurrent event analyses, follow-up was not censored at the occurrence of the outcomes, allowing us to study the rates of recurrent events over time while otherwise applying the same criteria for determining the end of follow-up as in the main analysis. To compare the effects of stimulant and non-stimulant drugs, we emulated a head-to-head trial comparing the effects of starting stimulants (methylphenidate, amphetamine, dexamphetamine, and lisdexamfetamine) with starting non-stimulants (atomoxetine and guanfacine) on the outcomes of interest. Follow-up began at the initiation of ADHD drug treatment and ended according to the same criteria used in the main analysis. We used SAS 9.4 and R version 4.4.0 for all analyses and defined statistical significance as a two tailed P value of ≤0.05.

### Subgroup analyses and sensitivity analyses

We did subgroup analyses based on sex, age (children and youths (<25 years), adults (≥25 years)), and people with and without a history of events. To test the robustness of our findings, we further did the following sensitivity analyses. Firstly, we extended the grace period to six months after diagnosis to account for potential variations in clinical practice and patients’ adherence. Secondly, we allowed drug switches during follow-up by not censoring individuals who switched between ADHD drugs. This approach enabled us to estimate the causal contrast between starting drug treatment for ADHD within three months after diagnosis and sustaining any ADHD drug treatment (that is, allow switching between ADHD drugs) versus not starting drug treatment for ADHD during the follow-up. Thirdly, we applied negative outcome control to assess potential biases and residual confounding.^50^ We used type 1 diabetes as a negative outcome given that previous studies did not find any significant effect of ADHD drug treatment on glycaemic management for type 1 diabetes.^51^

### Patient and public involvement

As this is a register based study, we had no direct contact with patients or participants at any stage. However, public discourse, media coverage, and interactions with individuals affected by ADHD show that many patients and care givers lack awareness of the risks and benefits of ADHD drug treatment, leading to uncertainty in treatment decisions. This knowledge gap served as a key motivation for our research. We discussed the aim and design of this study with representatives of people with lived experience of ADHD from ADHD Europe, the largest association of people with lived experience of ADHD in Europe. The board of ADHD Europe noted the importance of this research and the need for evidence from routine clinical settings. Their feedback guided the selection of outcomes and informed the interpretation of the findings.

## Results

### Baseline characteristics of study populations

We identified 148 581 individuals with a new ADHD diagnosis (41.3% female; median age 17.4 (interquartile range 11.6-29.1) years) (fig 1; table 1). During the two year follow-up, 4502 individuals had suicidal behaviours, 17 347 had substance misuse, and 24 065 had accidental injuries. In those with ADHD diagnosed after age 15 (n=89 672; 49.8% female), 4345 had transport accidents and 11 248 had criminality (supplementary table D). Within three months of an ADHD diagnosis, 84 282 (56.7%) individuals started drug treatment for ADHD and 64 377did not (fig 1). During the grace period, 78 individuals who died or emigrated contributed to both treatment strategies. Methylphenidate was the most prescribed drug at initiation (74 515; 88.4%), followed by atomoxetine (6676; 7.9%) and lisdexamfetamine (2749; 3.3%). Table 1 shows baseline characteristics by treatment strategy; covariate balance after weighting between strategies was adequate (standardised mean difference <0.1; supplementary tables E-I**)**.

### ADHD drug treatment and first events

**Fig 2:**
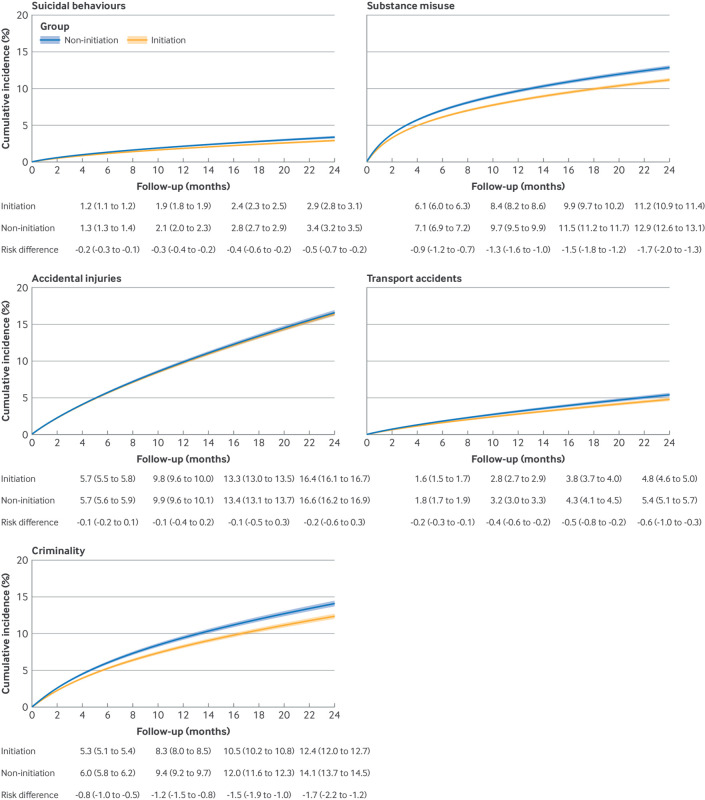
Cumulative incidence of first occurrence of outcomes over two years of follow-up: suicidal behaviours, substance misuse, accidental injuries, transport accidents, and criminality, all stratified by attention deficit/hyperactivity disorder drug treatment strategy. Numbers reported are weighted and account for follow-up censoring, including treatment discontinuation or switching

**Fig 3:**
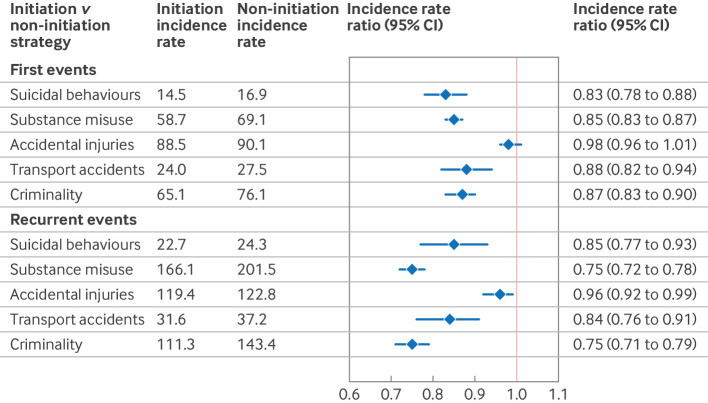
Attention deficit/hyperactivity disorder (ADHD) drug treatment and rates of first and recurrent outcome events over two years of follow-up among people with ADHD. Incidence rates were calculated per 1000 person years. Numbers reported are weighted and account for follow-up censoring, including treatment discontinuation or switching. CI=confidence interval

Figure 2 shows the cumulative incidence of the outcomes within two years after ADHD diagnosis in the initiation and non-initiation groups. ADHD drug treatment was associated with a statistically significant decreased rate of four of the five outcomes (fig 3): suicidal behaviours (weighted incidence rates 14.5 per 1000 person years in the initiation group versus 16.9 in the non-initiation group; adjusted incidence rate ratio 0.83, 95% confidence interval (CI) 0.78 to 0.88), substance misuse (incidence rate 58.7 *v* 69.1; incidence rate ratio 0.85, 0.83 to 0.87), transport accidents (incidence rate 24.0 *v* 27.5; incidence rate ratio 0.88, 0.82 to 0.94), and criminality (incidence rate 65.1 *v* 76.1; incidence rate ratio 0.87, 0.83 to 0.90). The estimates for accidental injuries were not statistically significant (incidence rate 88.5 *v* 90.1; incidence rate ratio 0.98, 0.96 to 1.01).

### ADHD drug treatment and recurrent events

**Fig 4:**
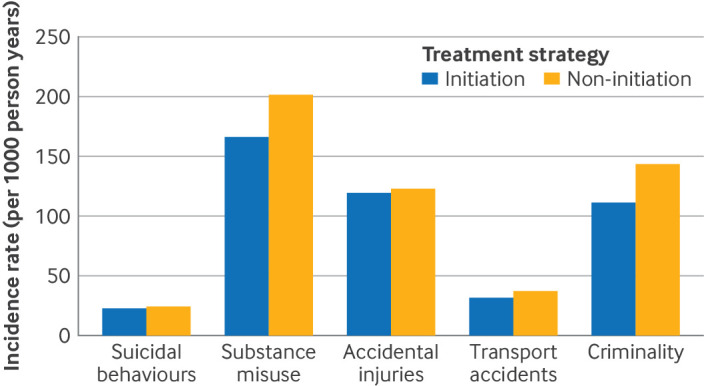
Incidence rates of recurrent events of outcomes over two years of follow-up. Incidence rates were calculated per 1000 person years. Numbers reported are weighted and account for follow-up censoring, including treatment discontinuation or switching

In the secondary analyses of recurrent events, ADHD drug treatment was associated with statistically significantly lower rates for all outcomes (fig 3), with incidence rate ratios of 0.85 (0.77 to 0.93) for suicidal behaviours, 0.75 (0.72 to 0.78) for substance misuse, 0.96 (0.92 to 0.99) for accidental injuries, 0.84 (0.76 to 0.91) for transport accidents, and 0.75 (0.71 to 0.79) for criminality. Figure 4 shows the weighted event rates in the initiation and non-initiation groups.

### Comparison between stimulants and non-stimulants

Stimulants were associated with lower event rates than non-stimulants, with incidence rate ratios ranging from 0.74 (95% CI 0.72 to 0.76) for substance misuse to 0.95 (0.93 to 0.98) for accidental injuries in the case of first events and from 0.71 (0.69 to 0.73) for criminality to 0.97 (0.95 to 0.99) for accidental injuries for recurrent events (supplementary table J).

### Subgroup analyses

**Fig 5:**
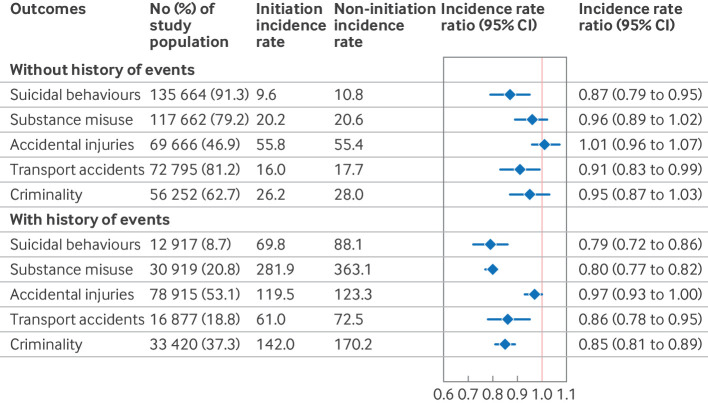
Attention deficit/hyperactivity disorder (ADHD) drug treatment and rates of first outcome event over two years of follow-up among individuals with ADHD, by history of events. Incidence rates were calculated per 1000 person years. Numbers reported are weighted and account for follow-up censoring, including treatment discontinuation or switching. Wald test for differences between incidence rate ratios for individuals without history versus those with history of events across five outcomes (suicidal behaviours, substance misuse, unintentional injuries, transport accidents, and criminality) yielded P values of 0.14, <0.01, 0.22, 0.40, and 0.02, respectively. CI=confidence interval

Of the study population, 12 917 (8.7%) had previous suicidal behaviour, 30 919 (20.8%) had previous substance misuse, 78 915 (53.1%) had previous accidental injury, 16 877 (18.8%) had previous transport accidents, and 33 420 (37.3%) had previous criminality (fig 5). Among people without a previous event, ADHD drug treatment was linked to reduced rates of suicidal behaviours (incidence rate ratio 0.87, 95% CI 0.79 to 0.95) and transport accidents (0.91, 0.83 to 0.99). By contrast, for those with a previous event, reductions were more pronounced and significant across all outcomes, with incidence rate ratios ranging from 0.79 (0.72 to 0.86) for suicidal behaviours to 0.97 (0.93 to 1.00) for accidental injuries. The risk reduction was statistically stronger for those with a history of substance misuse (P<0.01) and criminality (P=0.02) than for those without such a history (fig 5 **)**.

When we examined the associations by sex and age, rate reductions were more pronounced in adults than in children and youths for substance misuse (incidence rate ratio 0.83 (95% CI 0.80 to 0.86) *v* 0.92 (0.88 to 0.96); P<0.01) and criminality (incidence rate ratio 0.81 (0.77 to 0.85) *v* 0.90 (0.85 to 0.95); P<0.01) and more pronounced in female patients than in male patients for criminality (incidence rate ratio 0.81 (0.74 to 0.87) *v* 0.90 (0.86 to 0.94; P<0.01) (supplementary tables K and L). For recurrent events, the rate reduction was significant for suicidal behaviours (incidence rate ratio 0.80, 0.70 to 0.91) in children and youths but not in adults (incidence rate ratio 0.96, 0.80 to 1.10) (supplementary table M). We found no significant sex differences for recurrent outcomes (supplementary table N).

### Sensitivity analyses

**Fig 6:**
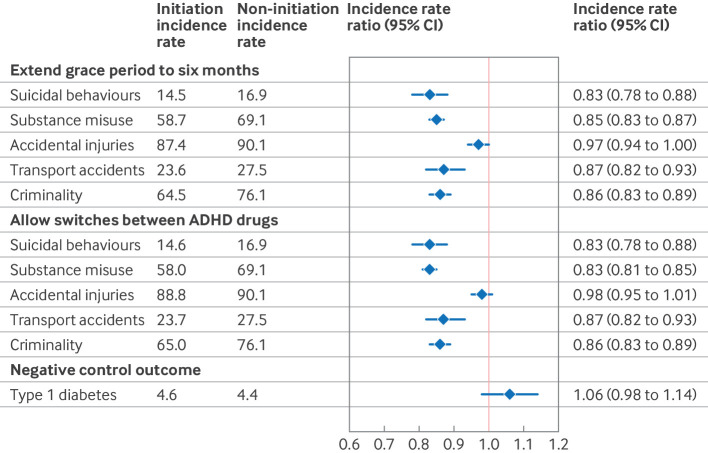
Sensitivity analyses of attention deficit/hyperactivity disorder drug treatment and rates of first outcome event over two years of follow-up. Incidence rates were calculated per 1000 person years. Numbers reported are weighted and account for follow-up censoring. CI=confidence interval

In sensitivity analyses, extending the grace period to six months or allowing switches between ADHD drugs during follow-up showed associations between use of ADHD drugs and rates of first event similar to the main analysis (fig 6). In the negative control analysis, we observed no statistically significant association between ADHD drug treatment and type 1 diabetes (incidence rate ratio 1.06, 95% CI 0.98 to 1.14; fig 6), suggesting that the risk of bias from unmeasured confounding (for example, greater health awareness, social engagement, and support) is unlikely to explain the associations between treatment and studied outcomes.

## Discussion

In these emulated trials using a nationwide ADHD sample, we found for the first time that drug treatment for ADHD was associated with reduced rates of a first occurrence of suicidal behaviours, substance misuse, transport accidents, and criminality over two years of follow-up. The estimate for first occurrence of accidental injuries was not statistically significant; however, when we considered recurrent events, ADHD drug treatment was statistically associated with a reduced rate of all five outcomes. Additionally, ADHD drug treatment was associated with greater risk reduction in people with a history of the outcome event and for repeated suicidal behaviour events in children and youths. Stimulant drugs were associated with lower rates of all five outcomes compared with non-stimulant drugs.

### Comparison with previous studies

The beneficial effects of ADHD drug treatment observed in our study may be explained by reductions in impulsivity and improvements in attention and executive functions, in line with findings from randomised controlled trials.^14^ ^52^ For instance, reduced impulsivity may lower criminality by curbing aggressive behaviour, whereas enhanced attention may decrease the risk of transport accidents by minimising distractions. These findings are consistent with those of previous observational studies using within individual designs.^17^ ^18^ ^19^ ^20^ ^21^ ^22^ ^23^ ^24^ However, the magnitude of rate reduction observed in our study is smaller. For suicidal behaviour, a meta-analysis of within individual studies reported a 31% reduction,^53^ whereas we observed a 15% rate reduction in recurrent suicidal events. Similarly, previous studies found reductions in criminality ranging from 32% to 41%,^24^ whereas our results showed a 25% rate reduction in recurrent events. For accidental injuries, previous meta-analysis reported a 12% rate reduction,^21^ compared with a 4% reduction in recurrent events in our data. Although we found no significant association for first accidental injuries, the modest reduction in repeated accidental injury rates remains clinically relevant, given their high prevalence (more than 16% of the sample affected during follow-up). Overall, the smaller effects observed in our study may partly reflect differences in study design. Unlike previous within individual studies focusing only on people with ADHD drug treatment who experienced events,^54^ our emulated trials compared initiators and non-initiators across the full ADHD population, providing average treatment effects more reflective of the entire patient population and closer to estimates expected from randomised controlled trials.^13^ ^55^ ^56^

The increasing use of drug treatment for ADHD over the past decades,^6^ ^7^ particularly notable among adult and female patients,^6^ ^7^ has likely led to the inclusion of individuals with fewer impairments and a less severe ADHD population,^57^ which may also contribute to the smaller effect sizes observed in our study. We found similar reduced risks among male and female patients, consistent with previous research.^17^ ^18^ The only notable exception was a stronger reduction in first crime convictions among female patients, although we observed no significant sex difference in analyses of recurrent events. Whereas male patients with ADHD have a higher absolute risk of criminal convictions, previous studies suggest that female patients have a higher relative risk,^58^ ^59^ potentially contributing to the stronger association with criminality among female patients shown in our study.

Many people with ADHD experience adverse outcome events multiple times. We found that the rate reductions associated with use of ADHD drug treatment were more pronounced for recurrent events than for first occurrences. This may be because people with multiple occurrences of such events typically have more severe ADHD, making them more likely to benefit from drug treatment.^60^ This is further supported by our analyses in individuals with a previous history of events. Additionally, the cumulative effect of ADHD drug treatment may lead to additive improvements over time,^61^ whereas negative consequences may accumulate the longer an individual goes untreated.^62^ ^63^ Together, these factors likely account for the greater rate reduction observed for recurrent events than for first occurrences in our study. This pattern also suggests that ADHD drug treatment may be associated with a true reduction in event rates rather than simply postponing the occurrence of these outcomes.

The more pronounced effects of stimulants compared with non-stimulants that we observed are in line with evidence from randomised controlled trials and align with current clinical guidelines. Randomised controlled trials have shown that stimulants are generally more effective than non-stimulants in reducing core ADHD symptoms.^8^ Improved symptom control could, in turn, reduce the risk of adverse outcomes over time. This finding is consistent with most guidelines that generally recommend stimulants as the first line drug treatment, followed by non-stimulants.^64^ Our results strengthen this recommendation by providing supporting evidence from population based, routinely collected clinical data.

### Strengths and limitations of study

A key strength of this study is the use of national registers combined with the target trial emulation design, providing evidence representative of patients in routine clinical settings. Additionally, the broad age range allowed for the examination of associations in both children and adults. The robustness of our findings was supported through sensitivity analyses and the negative control analysis. However, the study has several limitations. Firstly, data on non-drug treatment were not available, so our comparisons reflect use of ADHD drug treatment relative to “care as usual,” which may include psychotherapy. Unlike in randomised controlled trials that typically compare ADHD drug treatment with placebo, this may lead to conservative estimates of treatment effects. Future research incorporating data on both drug and non-drug treatment is needed. Secondly, exposure misclassification is possible, as some individuals might not have consistently taken their treatment as prescribed, potentially biasing the association towards the null. Thirdly, we were unable to assess the impact of drug dosage, which can vary over time depending on individual response to and tolerability of ADHD drug treatment, introducing variability that our study could not account for. Fourthly, although register based data offer comprehensive national coverage, our analyses might not capture less severe outcomes that are not brought to medical or legal attention. Fifthly, data on the symptomatic predominance of ADHD (inattention, hyperactivity/impulsivity, or combined) were not available, limiting our ability to do subgroup analyses. However, given the limited longitudinal stability of these presentations and the lack of evidence for differential treatment response,^65^ ^66^ their clinical utility, particularly in informing treatment strategies and predicting treatment outcomes, remains a matter of ongoing debate. Sixthly, although we aimed to examine the causal effects of ADHD drug treatment on the outcomes by using a target trial emulation design, negative control, and multiple sensitivity analyses, residual confounding from unmeasured factors, such as severity of ADHD, genetic predispositions, and lifestyle factors, may still exist. Finally, the findings may not be generalisable to other settings owing to differences in access to healthcare, diagnostic criteria, and prescribing practices across populations; for example, in our study, 88.4% of users of ADHD drugs started with methylphenidate; this is similar to many European countries, but the treatment context may differ from other countries.^36^

### Clinical implications

This study provides evidence on the effects of starting and sustaining drug treatment for ADHD on important clinically relevant outcomes. These findings are applicable to people with ADHD in routine clinical settings, who face challenges across different domains and throughout different phases of their lives.^67^ ^68^ For example, youths with ADHD have high rates of self-harm (almost 13% in our study),^69^ highlighting the urgent need for effective interventions during this critical developmental stage. Additionally, our findings indicating that stimulants were associated with greater reductions than non-stimulants contribute to informing decision making in the selection of drug treatment in clinical practice. Furthermore, our results highlight the need for well powered, long term, and representative trials that assess outcomes beyond ADHD core symptoms, to ensure that clinical guidelines for ADHD based on such trials are applicable to the populations seen in routine practice. Meeting this need will require integrated research efforts, including pragmatic trials—that is, those nested within registries and administrative databases—that complement conventional randomised controlled trials by capturing diverse patient populations often excluded from them. Overall, our study provides relevant information on additional benefits that are not captured in current randomised controlled trials, offering valuable insights for patients, clinicians, guideline developers, and other stakeholders weighing the benefits and risks of treatment. For instance, these findings are particularly important in informing the ongoing discussion about the inclusion of methylphenidate in the World Health Organization’s model list of essential medicines.^70^

### Conclusion

In this nationwide study using a target trial emulation design, drug treatment for ADHD was associated with reduced rates of a first occurrence of suicidal behaviours, substance misuse, transport accidents, and criminality over a two year follow-up, whereas the estimate for accidental injuries was not statistically significant. For recurrent events, ADHD drug treatment was statistically associated with reduced rates of all these outcomes, including accidental injuries. The observed reduced rates were more pronounced among patients with a history of outcome events and for stimulants versus non-stimulants. These results provide evidence on the effects of ADHD drug treatment on important health related and social outcomes that should inform clinical practice and the debate on the drug treatment of ADHD.

## What is already known on this topic

Attention deficit/hyperactivity disorder (ADHD) is associated with many adverse outcomes (eg, suicidal behaviours, substance misuse, accidental injuries, transport accidents, and criminality)
Many people with ADHD experience adverse outcome events multiple times
Randomised controlled trials have not evaluated the effects of ADHD drug treatment on broader clinical outcomes and may have limited generalisability to the entire ADHD population

## What this study adds

ADHD drug treatment was associated with significantly reduced rates of first occurrences of suicidal behaviours, substance misuse, transport accidents, and criminality but not accidental injuries
For recurrent events, ADHD drug treatment was statistically associated with reduced rates of all five outcomes
This is the first target trial emulation study showing beneficial effects of ADHD drug treatment on broader clinical outcomes in the entire ADHD population

## Data Availability

No additional data available. The Public Access to Information and Secrecy Act in Sweden prohibits individual level data being publicly available. Researchers who are interested in replicating this study can apply for individual level data through Statistics Sweden (https://www.scb.se/en/services/ordering-data-and-statistics/ordering-microdata/) and the National Board of Health and Welfare (https://www.socialstyrelsen.se/en/statistics-and-data/registers/). The underlying code is freely available at https://osf.io/y7fhj/.

## Web extra

Extra material supplied by authors

Web appendix: Supplementary materials

## Notes

Contributors: LZ, HL, SC, and ZC conceived and designed the study. LZ analysed the data and drafted the manuscript. NZ, AS, MN, MG, and LL assisted with the methods and analysis. RKH, IB, PL, HL, BMD, and ZC were involved in data curation. All authors commented on the draft and contributed to the writing of the final manuscript and interpretation of the data. ZC and SC contributed equally to this work and are the joint last authors. LZ, SC, and ZC are the guarantors. The corresponding author attests that all listed authors meet authorship criteria and that no others meeting the criteria have been omitted.

Transparency: The lead author (the manuscript’s guarantor) affirms that the manuscript is an honest, accurate, and transparent account of the study being reported, that no important aspects of the study have been omitted, and that any discrepancies from the study as planned (and, if relevant, registered) have been explained.

Dissemination to participants and related patient and public communities: To ensure broad dissemination, we will engage with ADHD Europe and issue press releases jointly with CHADD, EUNETHYDIS, APSARD, Karolinska Institutet, and coauthors’ institutions and share findings via social media (eg, X, LinkedIn). By leveraging these channels, we aim to reach a diverse audience, including patients, care givers, clinicians, policy makers, and other stakeholders, and contribute to informed decision making in ADHD treatment.

Provenance and peer review: Not commissioned; externally peer reviewed.
