# ADHD drug treatment and risk of suicidal behaviours, substance misuse, accidental injuries, transport accidents, and criminality: emulation of target trials

**DOI:** 10.1136/bmj.r1827

**Published:** 2025-09-01

In this paper by Zhang and colleagues (BMJ 2025;390:e083658, doi:10.1136/bmj-2024-083658, published 13 August 2025), the groups were mislabelled in figure 2. In fact, the blue line represents the non-initiation group, and the yellow line represents the initiation group. This has now been corrected.

