# Supplementary material for: ADHD drug treatment and risk of suicidal behaviours, substance misuse, accidental injuries, transport accidents, and criminality: emulation of target trials
Source: BMJ. 2025 Aug 13;390:e083658. doi: 10.1136/bmj-2024-083658 (PMC12344785; doi:10.1136/bmj-2024-083658)
Supplement: Supplementary file 1 — Web appendix: Supplementary materials [file zhle083658.ww1.pdf]

Supplementary material

Supplementary Methods.

Target trial emulation using cloning, censoring, and weighting.

Supplementary Figures

Figure A. Directed Acyclic Graph (DAG) illustrating the causal relationship between ADHD medication use and the outcomes.

Figure B. Schematic representation of cloning, censoring, and weighting method.

Supplementary Tables

Table A. Protocol of target trials on ADHD medication on first and recurrent event of five outcomes in individuals with ADHD and emulation using observational data.

Table B. Definitions of outcomes.

Table C. International Classification of Diseases (ICD) codes and Anatomical Therapeutic Chemical (ATC) codes for covariates.

Table D. Number of events and crude incidence rates over 2 years of follow-up among individuals with ADHD.

Table E. Covariate balance at the end of grace period before and after weighting for suicidal behaviours.

Table F. Covariate balance at the end of grace period before and after weighting for substance misuse.

Table G. Covariate balance at the end of grace period before and after weighting for accidental injuries.

Table H. Covariate balance at the end of grace period before and after weighting for transport accidents.

Table I. Covariate balance at the end of grace period before and after weighting for criminality.

Table J. ADHD medication and first and recurrent event rates over 2 years of follow-up among individuals with ADHD, stimulants vs non-stimulants

Table K. ADHD medication and rates of first event over 2 years of follow-up among individuals with ADHD, by age groups.

Table L. ADHD medication and rates of first event over 2 years of follow-up among individuals with ADHD, by sex.

Table M. ADHD medication and rates of recurrent events over 2 years of follow-up among individuals with ADHD, by age groups.

Table N. ADHD medication and rates of recurrent events over 2 years of follow-up among individuals with ADHD, by sex.

Supplementary Methods

Target trial emulation using cloning, censoring, and weighting

Here we describe in detail our implementation of the cloning, censoring, and weighting approach to compare the strategies "starting drug treatment for ADHD within three months after diagnosis and remaining on the prescribed drug" versus "not starting drug treatment for ADHD during the follow-up" in individuals with ADHD. This approach was used to estimate the effects of sustained treatment on five clinically important outcomes: suicidal behaviours, substance misuse, accidental injuries, transport accidents, and criminality.

1. Cloning step

In the cloning step, we created two identical copies (clones) of each eligible individual at baseline. One copy was assigned to the strategy of starting ADHD medication within three months of diagnosis and remaining on treatment (initiation group), while the other was assigned to the strategy of not starting ADHD medication during follow-up (non-initiation group). This expanded the dataset to twice its original size and ensured alignment of eligibility, treatment assignment, and start of follow-up. Because each individual appeared in both treatment arms, this step removed baseline confounding and prevented immortal time bias.

Cloning emulates randomization by assigning each individual to both treatment strategies at time zero, mimicking a randomized trial where treatment is assigned at baseline.[1 2] Combined with a grace period, it aligns treatment assignment and follow-up, preventing exposure misclassification and accounting for natural delays in treatment initiation.[3] Without cloning, methods like time-varying exposures or inverse probability weighting alone may misalign treatment and follow-up, leading to biased estimates. Cloning also enables estimation of marginal treatment effects by including all individuals in both arms, improving the robustness and generalizability of results.

2. Censoring step

In the censoring step, we assessed adherence to the assigned treatment strategy at monthly intervals (30-day). Then copies are artificially censored if and when they deviate from their assigned treatment strategy, which ensures that the copies follow their assigned strategy. Initiation of ADHD medication on the same day as the diagnosis was considered "initiating treatment on day 1." Copies assigned to the initiation arm would be censored at the third month if they did not have any dispensation of ADHD medication before/on day 90, or would be censored at the month when they discontinued ADHD medication treatment or switched to another ADHD medication after the grace period. Copies assigned to the non-initiation arm would be censored at the corresponding month when they had a dispensation of ADHD medication during the follow-up. Each individual's treatment strategy was completely determined at the end of the grace period, so at most only one copy from each individual still contributes person-time to the analysis by the end of the grace period.

3. Weighting step

In the weighting step, we applied inverse probability of censoring weighting to account for potential selection bias introduced by artificial censoring. Informally, uncensored copies receive a weight equal to the inverse of the probability of remaining uncensored, conditional on their history of treatment and

covariates. Intuitively, copies who are censored transfer their weights in the analysis to those who remain uncensored. This creates a hypothetical population in which censoring is independent of measured covariates.

We estimated time-varying inverse probability weights using separate pooled logistic regression models for each treatment arm.[3 4] These models included time (modeled with natural cubic splines) and all time-fixed and time-varying covariates described in the manuscript. Time-fixed confounders included age (continuous), calendar year (each year as a category), sex (male, female), birth country (Sweden, countries other than Sweden), highest education level (primary or lower secondary, upper secondary, post-secondary or postgraduate, unknown; using parents' highest education level for those younger than 25 years), number of outpatient visits (0, 1-4, 5-9, 10+) for psychiatric and non-psychiatric reasons, number of hospitalizations (0, 1-2, 3-4, 5+) for psychiatric and non-psychiatric reasons, diagnosis of psychiatric disorders (anxiety disorder, autism spectrum disorder, bipolar disorder, conduct disorder, depressive disorder, eating disorder, intellectual disability, personality disorder, schizophrenia, alcohol use disorder, , and substance use disorder), physical diseases (cardiovascular disease, epilepsy, type 2 diabetes, and hyperlipidaemia), history of the outcome event (suicidal behaviours, substance misuse, accidental injuries, transport accidents, or criminality), and dispensation of other psychotropic medications (antipsychotics, anxiolytics, hypnotics, and sedatives, antidepressants, antiepileptic drugs, anti-addiction drugs, and opioid). Time-varying confounders included the abovementioned diagnoses, dispensations, any outpatient visit for psychiatric and non-psychiatric reasons, and any hospitalization for psychiatric and non-psychiatric reasons in the previous month.

With the exception of education level, which had 0.8% missing data, there were no missing values in covariates. We created a separate “unknown” category for missing education data, allowing these individuals to be included in the analysis as a separate category.

The summary statistics of the IPCWs (before truncation) at the end of the grace period were as follows: Mean: 1.33, Min: 1.00, Max: 2.91, Standard deviation: 0.30. After truncation of the IPCWs at the 99.5th percentile, the mean was 1.33 (Min: 1.00, Max: 1.98) with standard deviation of 0.29.

4. Primary analysis

We fitted an inverse probability weighted discrete-time hazard model using pooled logistic regression separately for each of the five outcomes. In the model, the outcome of interest was regressed on the treatment arm and time. Because the outcome of the models is rare within each interval (e.g., each month), the odds ratio from this model approximates the rate ratio.[5 6] To estimate the cumulative incidence of the outcomes, we used the regression-then-marginalization approach. First, we fitted a weighted outcome regression using a pooled logistic regression model, with treatment arm, time, and treatment-time interactions as independent variables. Second, we estimated the standardized (marginal) survival at month m for the initiation group (and also for non-initiation group), by multiplying the predicted conditional probabilities of remaining event-free through each month $t \leq$ _m given survival through $t-1$ for each study participant, and then averaging the estimate across all participants. Cumulative incidence of each outcome was computed as 1 minus the standardized survival. From the cumulative incidence curve, we obtained the absolute risks for initiators versus non-initiators for each of the five outcomes. For all the estimates, 95% confidence intervals (CIs) were computed using non-parametric bootstrap with 500 copies.

5. Secondary analysis

In secondary analyses, we examined the association between ADHD medication and recurrent events of the five outcomes. To minimise the risk of misclassifying follow-up visits or administrative codes as new events, we allowed a maximum of one event per individual per month. We extended the cloning, censoring, and weighting approach from the primary analysis to accommodate recurrent events. As in the primary analysis, each eligible individual was cloned and assigned to one of the two treatment strategies at baseline. Censoring rules remained the same. However, unlike the primary analysis, follow-up time was not censored after the first occurrence of an outcome, allowing individuals to continue contributing person-time to subsequent event intervals. This approach enabled us to estimate the rate of recurrence over time while maintaining the same methodological structure. Inverse probability of censoring weights was recalculated to account for continued follow-up beyond the first event, using the same covariate set and model structure as in the primary analysis. These weights adjusted for time-varying confounding and potential selection bias introduced by artificial censoring due to non-adherence. All other methodological aspects—including outcome modelling, bootstrapping for confidence intervals, and the use of weighted pooled logistic regression—were consistent with those used in the primary analysis.

How the cloning, censoring, and weighting design addresses immortal time bias

The cloning, censoring, and weighting approach avoids immortal time bias by ensuring that all individuals are followed from the same starting point, regardless of treatment initiation. In traditional observational studies, individuals who eventually receive treatment must survive long enough to do so, creating an artificial survival advantage for the treatment group.[7] By maintaining proper follow-up alignment with treatment assignment and eligibility criteria, we avoided this bias.[1] Additionally, by cloning individuals into both treatment strategies at time zero and censoring the untreated clone upon initiation, we prevented misclassification of exposure time, ensuring that only actual treatment periods contribute to the analysis. The clone method with a grace period for treatment allocation adjusts for bias due to the time difference between treatment initiation and ADHD diagnosis and accounts for natural variation in treatment initiation.[2] Furthermore, individuals who died or emigrated during the grace period contributed to both treatment strategies. This prevented bias that could arise if such individuals were only included in the untreated group, which would artificially favour the treatment group, distorting the true effect of ADHD medication.

References

1. Hernán MA, Sauer BC, Hernández-Díaz S, et al. Specifying a target trial prevents immortal time bias and other self-inflicted injuries in observational analyses. *J Clin Epidemiol* 2016;79:70-75. doi:
2. Xie Y, Bowe B, Al-Aly Z. Molnupiravir and risk of hospital admission or death in adults with covid-19: emulation of a randomized target trial using electronic health records. *BMJ* 2023;380:e072705.
3. Maringe C, Benitez Majano S, Exarchakou A, et al. Reflection on modern methods: trial emulation in the presence of immortal-time bias. Assessing the benefit of major surgery for elderly lung cancer patients using observational data. *International Journal of Epidemiology* 2020;49(5):1719-29.
4. Danaei G, García Rodríguez LA, Cantero OF, et al. Electronic medical records can be used to emulate target trials of sustained treatment strategies. *J Clin Epidemiol* 2018;96:12-22.
5. Emilsson L, García-Albéniz X, Logan RW, et al. Examining Bias in Studies of Statin Treatment and Survival in Patients With Cancer. *JAMA Oncol* 2018;4(1):63-70.
6. Greenland S. Quantitative methods in the review of epidemiologic literature. *Epidemiol Rev* 1987;9:1-30.

7. Lévesque LE, Hanley JA, Kezouh A, et al. Problem of immortal time bias in cohort studies: example using statins for preventing progression of diabetes. *BMJ* 2010;340:b5087.

Supplementary figure A. Directed Acyclic Graph (DAG) illustrating the causal relationship between ADHD medication use and the outcomes.

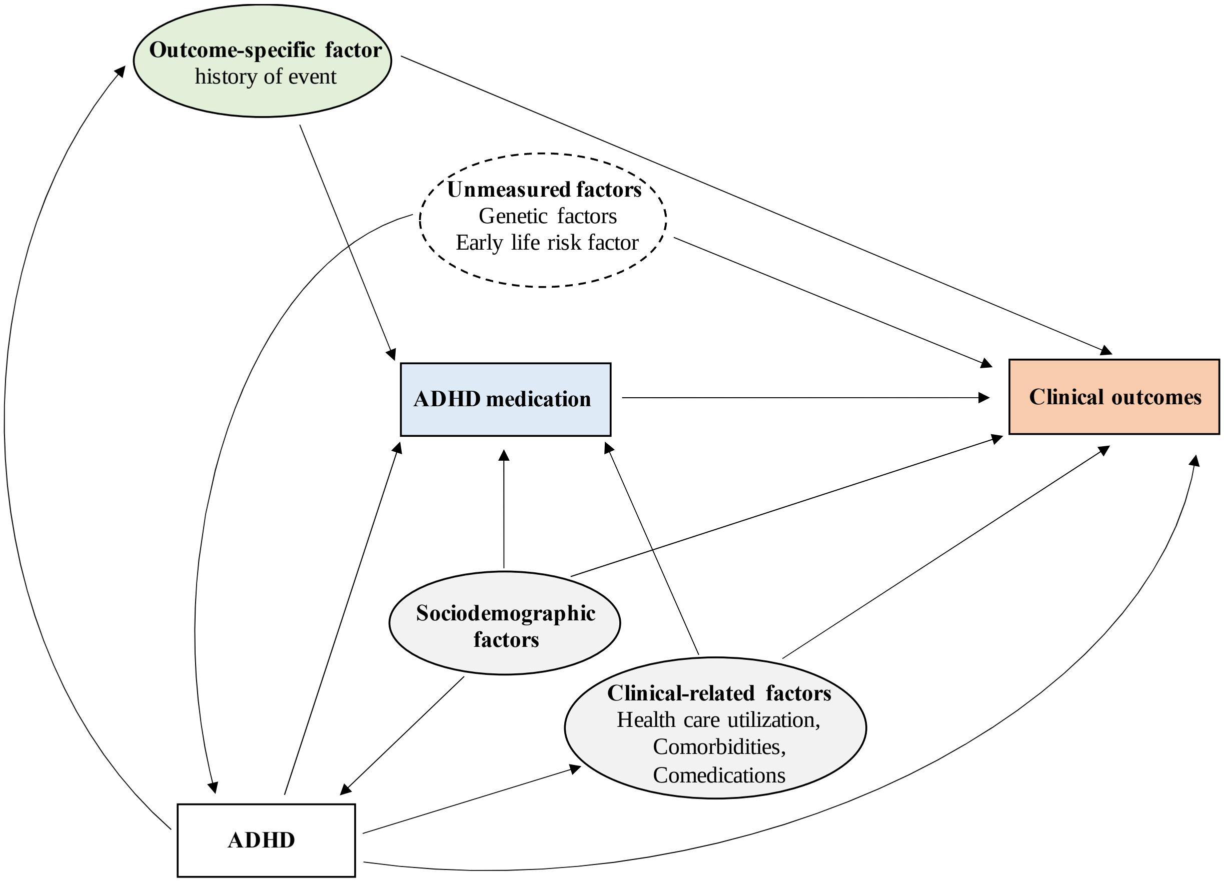

Directed Acyclic Graph (DAG) illustrating the hypothesized relationships between ADHD, medication use, and clinical outcomes. The DAG outlines assumed causal pathways and identifies potential confounders of the association between ADHD medication and each of the five adverse outcomes (suicidal behaviours, substance misuse, accidental injuries, transport accidents, and criminality). These confounders include socioeconomic and demographic variables (e.g., education, sex, age, birth country), clinical-related factors (e.g., healthcare utilization, psychiatric and physical comorbidities, comedications), outcome-specific factors (e.g., prior history of the specific outcome), and unmeasured variables (e.g., genetic predispositions). Arrows represent hypothesized causal directions. The DAG informed the selection of baseline and time-varying covariates included in the inverse probability weighting models used in the cloning, censoring, and weighting framework, thereby supporting confounding control and the emulation of target trials.

Supplementary figure B. Schematic representation of the cloning, censoring, and weighting method.

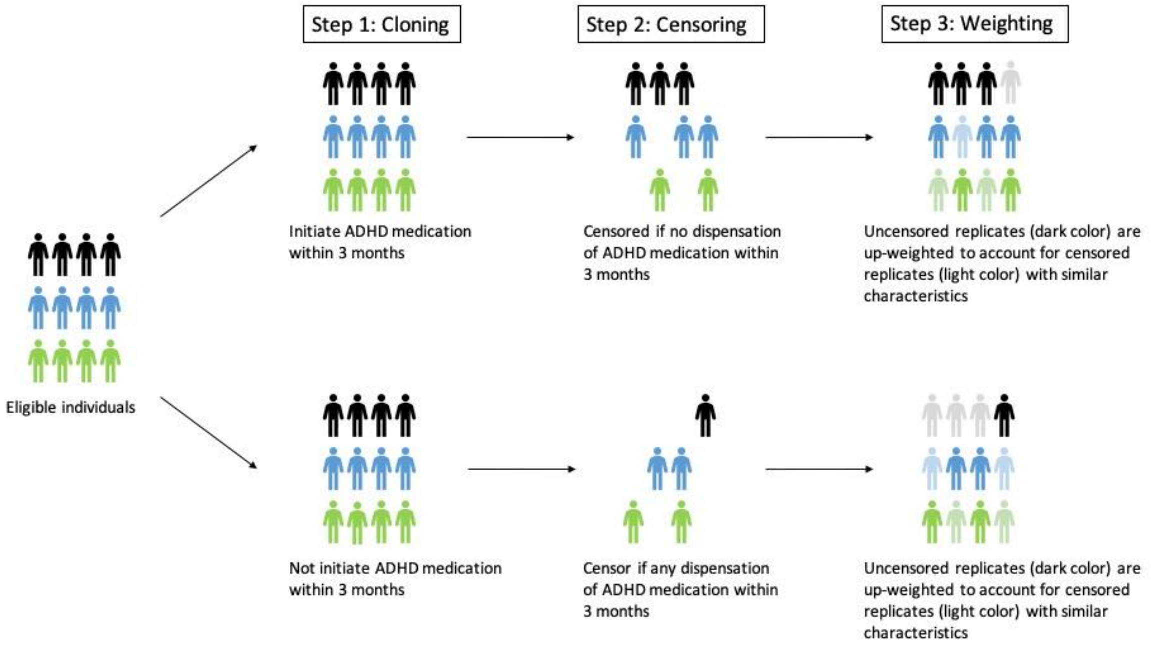

Supplementary table A. Protocol of target trials on ADHD medication on first and recurrent event of five outcomes in individuals with ADHD and emulation using observational data.

Protocol component	Target randomized trial	Emulation using observational data
**Eligibility criteria**	• Individuals (6-64 years) with an incident clinical diagnosis of ADHD between January 2007 and December 2018;<br>• No prior dispensation of ADHD medication.	• Same as the target trial.
**Treatment strategies**	• Starting drug treatment for ADHD within three months after diagnosis and remaining on the prescribed drug;<br>• Not starting drug treatment for ADHD during the follow-up.	• Same as the target trial.
**Assignment procedures**	Eligible individuals are randomly assigned to either strategy at baseline, and are aware of the assigned strategy.	Randomization is emulated by cloning eligible individuals and assigning each copy to one treatment strategy.
**Follow-up period**	Follow-up starts at treatment assignment and ends at an outcome of interest (only in time-to-first-event analysis), death, loss to follow-up (emigration), or administrative end of follow-up (December 31, 2020, or two years after baseline), whichever occurs first.	Same as the target trial.
**Outcomes**	Suicidal behaviours, substance misuses, accidental injuries, transport accidents and crime convictions within two years after baseline.	Same as the target trial.
**Causal contrasts of interest**	Effect of adhering to the treatment strategies (per-protocol) during follow-up.	Observational analogue of the per-protocol effect.
**Analysis plan**	Effect estimates are calculated from an inverse probability weighted pooled logistic regression model, with censoring at deviation from the protocol and adjustment for baseline and post-baseline covariates.	Same as the target trial, except that the analyses are conducted in an expanded dataset that includes copies of each eligible individual.

Supplementary table B: Definitions of outcomes.

Outcomes definition	Data sources and ICD-10 codes
**Suicidal behaviours**	From the Patient Register and Cause of Death Register: any of the following diagnoses: X60-X84, Y10-Y34
**Substance misuse**	From the Patient Register and Cause of Death Register: any of the following diagnoses: F10-F19, T36-T51, X40-X49<br>From the Crime Register: narcotics offence
**Accidental injuries**	From the Patient Register and Cause of Death Register: any of the following diagnoses: V, W, X00-X59
**Transport accidents**	From the Patient Register and Cause of Death Register: any of the following diagnoses: V01-V99<br>From the Crime Register: driving under the influence
**Criminality**	From the Crime Register: any type of convictions, including violent crimes (homicide, assault, robbery, arson, any sexual offence, illegal threats, or intimidation) and non-violent crimes.

Supplementary table C. International Classification of Diseases (ICD) codes and Anatomical Therapeutic Chemical (ATC) codes for covariates.

	ICD-10 codes/ATC codes
**Psychiatric comorbidities (ICD codes)**	
Alcohol use disorder	F10
Substance use disorder	F11-F16, F18-F19
Schizophrenia	F20-F29
Bipolar disorder	F30-F31
Depression	F32-F33
Anxiety disorders	F40-F41
Eating disorders	F50
Personality disorders	F60, F69
Intellectual disability	F70-F79
Autism spectrum disorders	F84
Conduct disorder	F91
**Other diseases and behaviours (ICD codes)**	
Cardiovascular disease	I00-I70, I73.0, I74-I75
Type 2 diabetes	E11
Dyslipidemia	E78
Epilepsy	G40-G41
**Psychotropic medications (ATC codes)**	
Antipsychotics	N05A
Anxiolytics, hypnotics, and sedatives	N05B, N05C
Antidepressants	N06A
Antiepileptic drugs	N03A
Anti-addiction drugs	N07B
Opioids	N02A

Supplementary table D. Number of events and crude incidence rates over 2 years follow-up among individuals with ADHD.

Outcome	Without treatment censoring[a]			With treatment censoring[b]		
	Events	Person-years	Incidence rate	Events	Person-years	Incidence rate
**Suicidal behaviours**	4,502	291,095	15.5	3,329	201,824	16.5
**Substance misuse**	17,347	273,127	63.5	14,053	190,113	73.9
**Accidental injuries**	24,065	269,434	89.3	17,355	188,795	91.9
**Transport accidents**	4,345	173,687	25.0	3,187	121,019	26.3
**Criminality**	11,248	164,790	68.3	8,676	115,209	75.3

Incidence rates were per 1,000 person-years.
a Follow-up ends at death, emigration, two years after baseline, or December 31st, 2020, whichever came first.
b Follow-up additionally ends at discontinuation or switch of ADHD medication.

Supplementary table E. Covariate balance at the end of grace period before and after weighting for suicidal behaviours.

		Before weighting			After weighting		
Characteristics	Overall	Initiation	Non-initiation	SMD	Initiation	Non-initiation	SMD
N	148581	83701	63825		148094.0	159317.3	
**Age at baseline (median, IQR)**	17.4 [11.6, 29.1]	16.4 [11.4, 27.8]	19.1 [11.9, 30.6]	0.14	16.6 [11.6, 28.2]	17.9 [11.3, 28.7]	0.03
**Sex**							
Male	87225 (58.7)	49396 (59.0)	37377 (58.6)	0.01	86359.1 (58.3)	93658.9 (58.8)	0.01
Female	61356 (41.3)	34305 (41.0)	26448 (41.4)	0.01	61734.9 (41.7)	65658.5 (41.2)	0.01
**Calendar year at baseline**				0.13			0.05
2007	4771 (3.2)	2095 (2.5)	2608 (4.1)		4144.4 (2.8)	5158.1 (3.2)	
2008	6500 (4.4)	3050 (3.6)	3360 (5.3)		5796.1 (3.9)	7031.8 (4.4)	
2009	7961 (5.4)	4175 (5.0)	3709 (5.8)		7421.4 (5.0)	8572.6 (5.4)	
2010	9842 (6.6)	5468 (6.5)	4263 (6.7)		9472.1 (6.4)	10634.3 (6.7)	
2011	11552 (7.8)	6731 (8.0)	4736 (7.4)		11371.8 (7.7)	12203.3 (7.7)	
2012	12709 (8.6)	7249 (8.7)	5384 (8.4)		12521.1 (8.5)	13619.8 (8.5)	
2013	13272 (8.9)	7580 (9.1)	5595 (8.8)		13257.6 (9.0)	14145.2 (8.9)	
2014	14463 (9.7)	8348 (10.0)	6023 (9.4)		14751.0 (10.0)	15301.2 (9.6)	
2015	15708 (10.6)	9160 (10.9)	6466 (10.1)		16177.1 (10.9)	16614.1 (10.4)	
2016	16508 (11.1)	9295 (11.1)	7126 (11.2)		16852.5 (11.4)	17988.1 (11.3)	
2017	17223 (11.6)	9867 (11.8)	7252 (11.4)		17640.3 (11.9)	18481.7 (11.6)	
2018	18072 (12.2)	10683 (12.8)	7303 (11.4)		18688.6 (12.6)	19567.2 (12.3)	
**Birth country**							
Sweden	136947 (92.2)	77675 (92.8)	58316 (91.4)	0.05	136910.4 (92.4)	147096.5 (92.3)	<0.01
Other[c]	11634 (7.8)	6026 (7.2)	5509 (8.6)	0.05	11183.6 (7.6)	12220.9 (7.7)	<0.01
**Education level at baseline[d]**				0.11			0.02
Primary or lower secondary	24784 (16.7)	12665 (15.1)	11879 (18.6)		23657.0 (16.0)	26517.4 (16.6)	
Upper secondary	75256 (50.6)	42664 (51.0)	32044 (50.2)		74903.8 (50.6)	80412.4 (50.5)	
Post-secondary or postgraduate	47340 (31.9)	27820 (33.2)	19279 (30.2)		48476.6 (32.7)	51187.8 (32.1)	
Unknown	1201 (0.8)	552 (0.7)	623 (1.0)		1056.6 (0.7)	1199.7 (0.8)	
**Comorbidities at baseline**							
Anxiety disorders	12086 (8.1)	6014 (7.2)	5883 (9.2)	0.07	11304.9 (7.6)	12791.1 (8.0)	0.01
Autism spectrum disorder	4771 (3.2)	2264 (2.7)	2444 (3.8)	0.06	4275.0 (2.9)	4786.4 (3.0)	0.01
Bipolar disorder	5142 (3.5)	2511 (3.0)	2534 (4.0)	0.05	4630.5 (3.1)	5454.7 (3.4)	0.02
Conduct disorder	2278 (1.5)	1356 (1.6)	872 (1.4)	0.02	2314.8 (1.6)	2365.7 (1.5)	0.01
Depressive disorder	28373 (19.1)	14879 (17.8)	12962 (20.3)	0.06	27137.4 (18.3)	30228.1 (19.0)	0.02
Eating disorder	3275 (2.2)	1696 (2.0)	1484 (2.3)	0.02	3135.7 (2.1)	3350.1 (2.1)	<0.01
Intellectual disability	2269 (1.5)	959 (1.1)	1290 (2.0)	0.07	1893.2 (1.3)	2308.3 (1.4)	0.01
Personality disorder	7233 (4.9)	3307 (4.0)	3691 (5.8)	0.09	6392.6 (4.3)	7518.9 (4.7)	0.02
Schizophrenia	2580 (1.7)	1020 (1.2)	1464 (2.3)	0.08	2092.3 (1.4)	2588.0 (1.6)	0.02
Epilepsy	3120 (2.1)	1306 (1.6)	1777 (2.8)	0.08	2657.8 (1.8)	3357.7 (2.1)	0.02
Alcohol use disorder	12351 (8.3)	6011 (7.2)	6005 (9.4)	0.08	11263.2 (7.6)	12837.8 (8.1)	0.02
Substance use disorder	12909 (8.7)	6075 (7.3)	6460 (10.1)	0.10	11666.1 (7.9)	13572.9 (8.5)	0.02
Cardiovascular disease	5005 (3.4)	2209 (2.6)	2720 (4.3)	0.09	4305.3 (2.9)	5299.6 (3.3)	0.02
Type 2 diabetes	1094 (0.7)	450 (0.5)	623 (1.0)	0.05	871.9 (0.6)	1172.4 (0.7)	0.02
Dyslipidemia	570 (0.4)	252 (0.3)	313 (0.5)	0.03	492.2 (0.3)	623.6 (0.4)	0.01
Suicidal behaviour	12459 (8.4)	6150 (7.3)	5832 (9.1)	0.07	11420.5 (7.7)	12731.0 (8.0)	0.01
**Psychotropic medication use at baseline**							
Opioids [e]	30496 (20.5)	16725 (20.0)	13407 (21.0)	0.03	29459.3 (19.9)	32726.0 (20.5)	0.02
Antiepileptic drugs	10743 (7.2)	5099 (6.1)	5409 (8.5)	0.09	9641.4 (6.5)	11417.4 (7.2)	0.03
Antipsychotics	14842 (10.0)	7275 (8.7)	7180 (11.2)	0.09	13502.6 (9.1)	15487.7 (9.7)	0.02
Anxiolytics, hypnotics, and sedatives	56763 (38.2)	30756 (36.7)	25248 (39.6)	0.06	55204.0 (37.3)	60504.7 (38.0)	0.01
Antidepressants	51506 (34.7)	27355 (32.7)	23419 (36.7)	0.08	49493.8 (33.4)	55047.8 (34.6)	0.02
Anti-addiction drugs [f]	7549 (5.1)	3785 (4.5)	3561 (5.6)	0.05	6810.3 (4.6)	7997.4 (5.0)	0.02
Number of prior hospitalizations				0.14			0.03

for psychiatric reason							
0	124644 (83.9)	72219 (86.3)	51997 (81.5)		126343.2 (85.3)	134281.8 (84.3)	
1-2	15306 (10.3)	7689 (9.2)	7372 (11.6)		14374.9 (9.7)	16220.7 (10.2)	
3-4	3639 (2.4)	1688 (2.0)	1848 (2.9)		3217.1 (2.2)	3813.3 (2.4)	
5+	4992 (3.4)	2105 (2.5)	2608 (4.1)		4158.9 (2.8)	5001.5 (3.1)	
Number of prior outpatient visit for psychiatric reason				0.12			0.02
0	81542 (54.9)	47974 (57.3)	33350 (52.3)		83269.5 (56.2)	88317.4 (55.4)	
1-4	38872 (26.2)	21478 (25.7)	17081 (26.8)		38401.0 (25.9)	41378.4 (26.0)	
5-9	13639 (9.2)	7053 (8.4)	6401 (10.0)		12973.3 (8.8)	14392.4 (9.0)	
10+	14528 (9.8)	7196 (8.6)	6993 (11.0)		13450.3 (9.1)	15229.1 (9.6)	
Number of prior hospitalizations for non-psychiatric reason				0.12			0.04
0	79491 (53.5)	46379 (55.4)	32808 (51.4)		81048.7 (54.7)	85372.2 (53.6)	
1-2	48535 (32.7)	27195 (32.5)	20986 (32.9)		48238.6 (32.6)	52042.0 (32.7)	
3-4	11534 (7.8)	6014 (7.2)	5356 (8.4)		10924.0 (7.4)	12354.8 (7.8)	
5+	9021 (6.1)	4113 (4.9)	4675 (7.3)		7882.7 (5.3)	9548.2 (6.0)	
Number of prior outpatient visits for non-psychiatric reason				0.05			0.01
0	25989 (17.5)	14429 (17.2)	11456 (17.9)		25590.7 (17.3)	27762.7 (17.4)	
1-4	65142 (43.8)	37421 (44.7)	27353 (42.9)		65715.1 (44.4)	69875.9 (43.9)	
5-9	31635 (21.3)	17983 (21.5)	13392 (21.0)		31629.9 (21.4)	33940.9 (21.3)	
10+	25815 (17.4)	13868 (16.6)	11624 (18.2)		25158.3 (17.0)	27737.8 (17.4)	
**Time varying covariates in the previous month**							
Anxiety disorders	1129 (0.8)	621 (0.7)	531 (0.8)	0.01	1184.7 (0.8)	1240.6 (0.8)	<0.01
Autism spectrum disorder	2824 (1.9)	1349 (1.6)	1265 (2.0)	0.03	2471.7 (1.7)	3051.9 (1.9)	0.02
Bipolar disorder	1179 (0.8)	541 (0.6)	549 (0.9)	0.02	1026.3 (0.7)	1215.6 (0.8)	0.01
Conduct disorder	282 (0.2)	195 (0.2)	96 (0.2)	0.02	341.0 (0.2)	254.8 (0.2)	0.02
Depressive disorder	2433 (1.6)	1333 (1.6)	1070 (1.7)	0.01	2407.6 (1.6)	2671.7 (1.7)	<0.01
Eating disorder	253 (0.2)	125 (0.1)	125 (0.2)	0.01	231.5 (0.2)	280.4 (0.2)	<0.01
Intellectual disability	381 (0.3)	186 (0.2)	181 (0.3)	0.01	360.3 (0.2)	382.5 (0.2)	<0.01
Personality disorder	910 (0.6)	392 (0.5)	427 (0.7)	0.03	769.5 (0.5)	922.8 (0.6)	0.01
Schizophrenia	319 (0.2)	110 (0.1)	171 (0.3)	0.03	228.4 (0.2)	304.7 (0.2)	0.01
Epilepsy	278 (0.2)	96 (0.1)	180 (0.3)	0.04	204.6 (0.1)	362.9 (0.2)	0.02
Alcohol use disorder	604 (0.4)	256 (0.3)	290 (0.5)	0.02	483.4 (0.3)	609.7 (0.4)	0.01
Substance use disorder	1265 (0.9)	552 (0.7)	662 (1.0)	0.04	1066.9 (0.7)	1413.3 (0.9)	0.02
Cardiovascular disease	350 (0.2)	151 (0.2)	159 (0.2)	0.01	282.6 (0.2)	325.9 (0.2)	<0.01
Type 2 diabetes	96 (0.1)	22 (0.0)	52 (0.1)	0.02	44.8 (0.0)	95.1 (0.1)	0.01
Dyslipidemia	18 (0.0)	9 (0.0)	11 (0.0)	0.01	17.8 (0.0)	22.7 (0.0)	<0.01
Suicidal behaviour	290 (0.2)	108 (0.1)	101 (0.2)	0.01	207.8 (0.1)	215.9 (0.1)	<0.01
Opioids [e]	3082 (2.1)	1596 (1.9)	1378 (2.2)	0.02	2858.2 (1.9)	3284.7 (2.1)	0.01
Antiepileptic drugs	3394 (2.3)	1491 (1.8)	1723 (2.7)	0.06	2858.6 (1.9)	3647.9 (2.3)	0.03
Antipsychotics	4769 (3.2)	2252 (2.7)	2230 (3.5)	0.05	4135.1 (2.8)	4791.4 (3.0)	0.01
Anxiolytics, hypnotics, and sedatives	19374 (13.0)	11419 (13.6)	7265 (11.4)	0.07	19501.7 (13.2)	18650.5 (11.7)	0.04
Antidepressants	13055 (8.8)	7083 (8.5)	5780 (9.1)	0.02	12730.2 (8.6)	13892.6 (8.7)	<0.01
Anti-addiction drugs [f]	781 (0.5)	374 (0.4)	372 (0.6)	0.02	684.4 (0.5)	822.3 (0.5)	0.01
Any hospitalizations for psychiatric reasons	1403 (0.9)	550 (0.7)	634 (1.0)	0.04	1058.4 (0.7)	1376.5 (0.9)	0.02
Any outpatient visits for psychiatric reasons	19501 (13.1)	11771 (14.1)	7708 (12.1)	0.06	20913.3 (14.1)	19410.9 (12.2)	0.06
Any hospitalizations for non-psychiatric reasons	995 (0.7)	400 (0.5)	452 (0.7)	0.03	746.0 (0.5)	986.0 (0.6)	0.02
Any outpatient visits for non-psychiatric reasons	7333 (4.9)	3885 (4.6)	3263 (5.1)	0.02	7006.1 (4.7)	7898.5 (5.0)	0.01

SMD, standardized mean difference. Data are numbers (%) unless stated otherwise. [a] Assessed at baseline. [b] Assessed at baseline. Those who died or emigrated and did not initiate ADHD medication during the grace period (n = 78) contributed to both treatment strategies. [c] Including all countries other than Sweden. [d] For those younger than 25 years, education level was replaced by parents' highest education level. [e] Refers to prescribed opioids in the Prescription Drug Register. [f] Including drugs used in nicotine dependence, drugs used in alcohol dependence, and drugs used in opioid dependence.

Supplementary table F. Covariate balance at the end of grace period before and after weighting for substance misuse.

		Before weighting			After weighting		
Characteristics	Overall	Initiation	Non-initiation	SMD	Initiation	Non-initiation	SMD
N	148581	81507	61043		142996.7	152561.1	
**Age at baseline (median, IQR)**	17.4 [11.6, 29.1]	16.1 [11.3, 27.1]	18.3 [11.6, 29.9]	0.13	16.3 [11.5, 27.4]	17.2 [11.0, 27.9]	0.02
**Sex**							
Male	87225 (58.7)	47895 (58.8)	35378 (58.0)	0.02	82859.6 (57.9)	88905.0 (58.3)	0.01
Female	61356 (41.3)	33612 (41.2)	25665 (42.0)	0.02	60137.2 (42.1)	63656.0 (41.7)	0.01
**Calendar year at baseline**				0.14			0.05
2007	4771 (3.2)	1990 (2.4)	2470 (4.0)		3891.4 (2.7)	4827.2 (3.2)	
2008	6500 (4.4)	2910 (3.6)	3158 (5.2)		5470.3 (3.8)	6565.5 (4.3)	
2009	7961 (5.4)	3998 (4.9)	3490 (5.7)		7049.6 (4.9)	7974.9 (5.2)	
2010	9842 (6.6)	5236 (6.4)	3995 (6.5)		8951.8 (6.3)	9964.3 (6.5)	
2011	11552 (7.8)	6460 (7.9)	4500 (7.4)		10817.0 (7.6)	11580.4 (7.6)	
2012	12709 (8.6)	6987 (8.6)	5113 (8.4)		11983.9 (8.4)	12943.5 (8.5)	
2013	13272 (8.9)	7400 (9.1)	5362 (8.8)		12805.3 (9.0)	13607.1 (8.9)	
2014	14463 (9.7)	8165 (10.0)	5759 (9.4)		14276.8 (10.0)	14691.6 (9.6)	
2015	15708 (10.6)	8977 (11.0)	6209 (10.2)		15707.6 (11.0)	15991.4 (10.5)	
2016	16508 (11.1)	9126 (11.2)	6889 (11.3)		16454.8 (11.5)	17433.2 (11.4)	
2017	17223 (11.6)	9714 (11.9)	7019 (11.5)		17253.7 (12.1)	17911.5 (11.7)	
2018	18072 (12.2)	10544 (12.9)	7079 (11.6)		18334.7 (12.8)	19070.6 (12.5)	
**Birth country**							
Sweden	136947 (92.2)	75652 (92.8)	55816 (91.4)	0.05	132262.0 (92.5)	140946.1 (92.4)	<0.01
Other[c]	11634 (7.8)	5855 (7.2)	5227 (8.6)	0.05	10734.7 (7.5)	11615.0 (7.6)	<0.01
**Education level at baseline[d]**				0.10			0.02
Primary or lower secondary	24784 (16.7)	11981 (14.7)	10933 (17.9)		22065.8 (15.4)	24405.6 (16.0)	
Upper secondary	75256 (50.6)	41541 (51.0)	30626 (50.2)		72281.9 (50.5)	76887.1 (50.4)	
Post-secondary or postgraduate	47340 (31.9)	27453 (33.7)	18882 (30.9)		47638.6 (33.3)	50107.9 (32.8)	
Unknown	1201 (0.8)	532 (0.7)	602 (1.0)		1010.4 (0.7)	1160.5 (0.8)	
**Comorbidities at baseline**							
Anxiety disorders	12086 (8.1)	5634 (6.9)	5374 (8.8)	0.07	10404.2 (7.3)	11595.4 (7.6)	0.01
Autism spectrum disorder	4771 (3.2)	2232 (2.7)	2383 (3.9)	0.07	4186.6 (2.9)	4678.0 (3.1)	0.01
Bipolar disorder	5142 (3.5)	2366 (2.9)	2351 (3.9)	0.05	4322.7 (3.0)	5059.2 (3.3)	0.02
Conduct disorder	2278 (1.5)	1319 (1.6)	823 (1.3)	0.02	2230.5 (1.6)	2244.7 (1.5)	0.01
Depressive disorder	28373 (19.1)	14092 (17.3)	12015 (19.7)	0.06	25350.7 (17.7)	27874.9 (18.3)	0.01
Eating disorder	3275 (2.2)	1647 (2.0)	1411 (2.3)	0.02	3000.7 (2.1)	3188.1 (2.1)	<0.01
Intellectual disability	2269 (1.5)	947 (1.2)	1251 (2.0)	0.07	1852.0 (1.3)	2240.1 (1.5)	0.01
Personality disorder	7233 (4.9)	3036 (3.7)	3262 (5.3)	0.08	5733.9 (4.0)	6614.5 (4.3)	0.02
Schizophrenia	2580 (1.7)	836 (1.0)	1214 (2.0)	0.08	1678.0 (1.2)	2073.3 (1.4)	0.02
Epilepsy	3120 (2.1)	1218 (1.5)	1639 (2.7)	0.08	2451.5 (1.7)	3081.4 (2.0)	0.02
Alcohol use disorder	12351 (8.3)	4929 (6.0)	4732 (7.8)	0.07	8913.1 (6.2)	9831.8 (6.4)	0.01
Substance use disorder	12909 (8.7)	4664 (5.7)	4727 (7.7)	0.08	8554.2 (6.0)	9508.6 (6.2)	0.01
Cardiovascular disease	5005 (3.4)	2038 (2.5)	2470 (4.0)	0.09	3948.5 (2.8)	4776.0 (3.1)	0.02
Type 2 diabetes	1094 (0.7)	408 (0.5)	564 (0.9)	0.05	790.1 (0.6)	1047.5 (0.7)	0.02
Dyslipidemia	570 (0.4)	234 (0.3)	291 (0.5)	0.03	451.2 (0.3)	569.9 (0.4)	0.01
Substance misuse	28395 (19.1)	12164 (14.9)	11234 (18.4)	0.09	22117.8 (15.5)	23752.6 (15.6)	<0.01
**Psychotropic medication use at baseline**							
Opioids [e]	30496 (20.5)	15689 (19.2)	12198 (20.0)	0.02	27202.0 (19.0)	29765.9 (19.5)	0.01
Antiepileptic drugs	10743 (7.2)	4732 (5.8)	4884 (8.0)	0.09	8765.8 (6.1)	10238.0 (6.7)	0.02
Antipsychotics	14842 (10.0)	6555 (8.0)	6229 (10.2)	0.08	11856.3 (8.3)	13282.6 (8.7)	0.01
Anxiolytics, hypnotics, and sedatives	56763 (38.2)	29126 (35.7)	23232 (38.1)	0.05	51457.7 (36.0)	55634.3 (36.5)	0.01
Antidepressants	51506 (34.7)	25869 (31.7)	21592 (35.4)	0.08	46084.3 (32.2)	50580.3 (33.2)	0.02
Anti-addiction drugs [f]	7549 (5.1)	2978 (3.7)	2668 (4.4)	0.04	5116.0 (3.6)	5791.4 (3.8)	0.01
Number of prior hospitalizations for psychiatric reason				0.12			0.02

0	124644 (83.9)	71396 (87.6)	51009 (83.6)		124392.4 (87.0)	131662.9 (86.3)	
1-2	15306 (10.3)	7086 (8.7)	6637 (10.9)		12960.0 (9.1)	14402.8 (9.4)	
3-4	3639 (2.4)	1483 (1.8)	1534 (2.5)		2732.1 (1.9)	3080.9 (2.0)	
5+	4992 (3.4)	1542 (1.9)	1863 (3.1)		2912.2 (2.0)	3414.4 (2.2)	
Number of prior outpatient visit for psychiatric reason				0.11			0.02
0	81542 (54.9)	47633 (58.4)	32845 (53.8)		82387.2 (57.6)	87068.0 (57.1)	
1-4	38872 (26.2)	20826 (25.6)	16263 (26.6)		36855.6 (25.8)	39321.3 (25.8)	
5-9	13639 (9.2)	6599 (8.1)	5861 (9.6)		11959.0 (8.4)	13081.0 (8.6)	
10+	14528 (9.8)	6449 (7.9)	6074 (10.0)		11795.0 (8.2)	13090.8 (8.6)	
Number of prior hospitalizations for non-psychiatric reason				0.11			0.03
0	79491 (53.5)	45745 (56.1)	32111 (52.6)		79605.5 (55.7)	83492.4 (54.7)	
1-2	48535 (32.7)	26409 (32.4)	19954 (32.7)		46324.7 (32.4)	49503.5 (32.4)	
3-4	11534 (7.8)	5670 (7.0)	4884 (8.0)		10119.8 (7.1)	11270.2 (7.4)	
5+	9021 (6.1)	3683 (4.5)	4094 (6.7)		6946.7 (4.9)	8294.9 (5.4)	
Number of prior outpatient visits for non-psychiatric reason				0.05			0.01
0	25989 (17.5)	14201 (17.4)	11168 (18.3)		25055.6 (17.5)	27023.4 (17.7)	
1-4	65142 (43.8)	36576 (44.9)	26283 (43.1)		63713.4 (44.6)	67207.1 (44.1)	
5-9	31635 (21.3)	17415 (21.4)	12676 (20.8)		30323.4 (21.2)	32215.5 (21.1)	
10+	25815 (17.4)	13315 (16.3)	10916 (17.9)		23904.4 (16.7)	26115.0 (17.1)	
**Time varying covariates in the previous month**							
Anxiety disorders	1129 (0.8)	595 (0.7)	488 (0.8)	0.01	1123.4 (0.8)	1133.5 (0.7)	<0.01
Autism spectrum disorder	2824 (1.9)	1332 (1.6)	1245 (2.0)	0.03	2423.4 (1.7)	3009.5 (2.0)	0.02
Bipolar disorder	1179 (0.8)	510 (0.6)	500 (0.8)	0.02	945.1 (0.7)	1108.6 (0.7)	0.01
Conduct disorder	282 (0.2)	193 (0.2)	92 (0.2)	0.02	333.3 (0.2)	245.6 (0.2)	0.02
Depressive disorder	2433 (1.6)	1297 (1.6)	1004 (1.6)	<0.01	2320.7 (1.6)	2518.2 (1.7)	<0.01
Eating disorder	253 (0.2)	123 (0.2)	121 (0.2)	0.01	225.2 (0.2)	271.7 (0.2)	0.01
Intellectual disability	381 (0.3)	180 (0.2)	179 (0.3)	0.01	348.1 (0.2)	377.1 (0.2)	<0.01
Personality disorder	910 (0.6)	364 (0.4)	398 (0.7)	0.03	696.0 (0.5)	843.0 (0.6)	0.01
Schizophrenia	319 (0.2)	96 (0.1)	133 (0.2)	0.02	193.1 (0.1)	230.2 (0.2)	<0.01
Epilepsy	278 (0.2)	88 (0.1)	167 (0.3)	0.04	187.5 (0.1)	336.1 (0.2)	0.02
Alcohol use disorder	604 (0.4)	156 (0.2)	170 (0.3)	0.02	283.3 (0.2)	343.8 (0.2)	0.01
Substance use disorder	1265 (0.9)	302 (0.4)	367 (0.6)	0.03	545.4 (0.4)	753.7 (0.5)	0.02
Cardiovascular disease	350 (0.2)	136 (0.2)	140 (0.2)	0.01	253.3 (0.2)	288.6 (0.2)	<0.01
Type 2 diabetes	96 (0.1)	18 (0.0)	46 (0.1)	0.02	36.6 (0.0)	83.9 (0.1)	0.01
Dyslipidemia	18 (0.0)	9 (0.0)	10 (0.0)	<0.01	17.3 (0.0)	21.0 (0.0)	<0.01
Substance misuse	2175 (1.5)	604 (0.7)	672 (1.1)	0.04	1102.0 (0.8)	1401.1 (0.9)	0.02
Opioids [e]	3082 (2.1)	1483 (1.8)	1245 (2.0)	0.02	2604.7 (1.8)	2957.3 (1.9)	0.01
Antiepileptic drugs	3394 (2.3)	1395 (1.7)	1593 (2.6)	0.06	2633.8 (1.8)	3347.8 (2.2)	0.03
Antipsychotics	4769 (3.2)	2065 (2.5)	1957 (3.2)	0.04	3684.8 (2.6)	4150.9 (2.7)	0.01
Anxiolytics, hypnotics, and sedatives	19374 (13.0)	10929 (13.4)	6676 (10.9)	0.08	18373.9 (12.8)	17138.7 (11.2)	0.05
Antidepressants	13055 (8.8)	6730 (8.3)	5394 (8.8)	0.02	11937.3 (8.3)	12921.3 (8.5)	<0.01
Anti-addiction drugs [f]	781 (0.5)	199 (0.2)	212 (0.3)	0.02	363.3 (0.3)	436.4 (0.3)	0.01
Any hospitalizations for psychiatric reasons	1403 (0.9)	458 (0.6)	487 (0.8)	0.03	841.6 (0.6)	1052.1 (0.7)	0.01
Any outpatient visits for psychiatric reasons	19501 (13.1)	11290 (13.9)	7182 (11.8)	0.06	19838.4 (13.9)	18153.6 (11.9)	0.06
Any hospitalizations for non-psychiatric reasons	995 (0.7)	357 (0.4)	387 (0.6)	0.03	652.2 (0.5)	850.0 (0.6)	0.01
Any outpatient visits for non-psychiatric reasons	7333 (4.9)	3698 (4.5)	3063 (5.0)	0.02	6603.6 (4.6)	7403.5 (4.9)	0.01

SMD, standardized mean difference. Data are numbers (%) unless stated otherwise. [a] Assessed at baseline. [b] Assessed at baseline. Those who died or emigrated and did not initiate ADHD medication during the grace period (n = 78) contributed to both treatment strategies. [c] Including all countries other than Sweden. [d] For those younger than 25 years, education level was replaced by parents' highest education level. [e] Refers to prescribed opioids in the Prescription Drug Register. [f] Including drugs used in nicotine dependence, drugs used in alcohol dependence, and drugs used in opioid dependence.

Supplementary table G. Covariate balance at the end of grace period before and after weighting for accidental injuries.

		Before weighting			After weighting		
Characteristics	Overall	Initiation	Non-initiation	SMD	Initiation	Non-initiation	SMD
N	148581	81806	62471		144938.4	155134.7	
**Age at baseline (median, IQR)**	17.4 [11.6, 29.1]	16.5 [11.4, 27.8]	19.1 [11.9, 30.6]	0.14	16.7 [11.6, 28.3]	17.9 [11.3, 28.7]	0.03
**Sex**							
Male	87225 (58.7)	48067 (58.8)	36455 (58.4)	0.01	84169.6 (58.1)	90839.9 (58.6)	0.01
Female	61356 (41.3)	33739 (41.2)	26016 (41.6)	0.01	60768.7 (41.9)	64294.8 (41.4)	0.01
**Calendar year at baseline**				0.14			0.05
2007	4771 (3.2)	2074 (2.5)	2568 (4.1)		4123.6 (2.8)	5057.2 (3.3)	
2008	6500 (4.4)	2984 (3.6)	3310 (5.3)		5698.3 (3.9)	6870.4 (4.4)	
2009	7961 (5.4)	4072 (5.0)	3637 (5.8)		7247.9 (5.0)	8358.6 (5.4)	
2010	9842 (6.6)	5358 (6.5)	4175 (6.7)		9307.3 (6.4)	10317.8 (6.7)	
2011	11552 (7.8)	6542 (8.0)	4636 (7.4)		11092.8 (7.7)	11888.7 (7.7)	
2012	12709 (8.6)	7074 (8.6)	5259 (8.4)		12246.0 (8.4)	13220.1 (8.5)	
2013	13272 (8.9)	7376 (9.0)	5457 (8.7)		12895.3 (8.9)	13736.2 (8.9)	
2014	14463 (9.7)	8143 (10.0)	5898 (9.4)		14395.3 (9.9)	14923.3 (9.6)	
2015	15708 (10.6)	8949 (10.9)	6312 (10.1)		15813.3 (10.9)	16187.6 (10.4)	
2016	16508 (11.1)	9066 (11.1)	6968 (11.2)		16460.8 (11.4)	17482.4 (11.3)	
2017	17223 (11.6)	9676 (11.8)	7098 (11.4)		17301.0 (11.9)	18029.4 (11.6)	
2018	18072 (12.2)	10492 (12.8)	7153 (11.5)		18356.7 (12.7)	19062.8 (12.3)	
**Birth country**							
Sweden	136947 (92.2)	75886 (92.8)	57081 (91.4)	0.05	133937.8 (92.4)	143232.2 (92.3)	<0.01
Other[c]	11634 (7.8)	5920 (7.2)	5390 (8.6)	0.05	11000.5 (7.6)	11902.5 (7.7)	<0.01
**Education level at baseline[d]**				0.10			0.02
Primary or lower secondary	24784 (16.7)	12384 (15.1)	11578 (18.5)		23175.7 (16.0)	25711.9 (16.6)	
Upper secondary	75256 (50.6)	41663 (50.9)	31339 (50.2)		73186.9 (50.5)	78299.5 (50.5)	
Post-secondary or postgraduate	47340 (31.9)	27212 (33.3)	18941 (30.3)		47526.6 (32.8)	49941.8 (32.2)	
Unknown	1201 (0.8)	547 (0.7)	613 (1.0)		1049.3 (0.7)	1181.5 (0.8)	
**Comorbidities at baseline**							
Anxiety disorders	12086 (8.1)	5935 (7.3)	5796 (9.3)	0.07	11168.2 (7.7)	12573.1 (8.1)	0.01
Autism spectrum disorder	4771 (3.2)	2248 (2.7)	2427 (3.9)	0.06	4265.5 (2.9)	4755.5 (3.1)	0.01
Bipolar disorder	5142 (3.5)	2492 (3.0)	2506 (4.0)	0.05	4617.2 (3.2)	5380.7 (3.5)	0.02
Conduct disorder	2278 (1.5)	1320 (1.6)	860 (1.4)	0.02	2264.8 (1.6)	2303.3 (1.5)	0.01
Depressive disorder	28373 (19.1)	14661 (17.9)	12810 (20.5)	0.07	26847.1 (18.5)	29767.3 (19.2)	0.02
Eating disorder	3275 (2.2)	1687 (2.1)	1488 (2.4)	0.02	3124.7 (2.2)	3347.4 (2.2)	<0.01
Intellectual disability	2269 (1.5)	937 (1.1)	1265 (2.0)	0.07	1870.5 (1.3)	2246.3 (1.4)	0.01
Personality disorder	7233 (4.9)	3281 (4.0)	3685 (5.9)	0.09	6388.6 (4.4)	7468.0 (4.8)	0.02
Schizophrenia	2580 (1.7)	1019 (1.2)	1460 (2.3)	0.08	2098.3 (1.4)	2562.2 (1.7)	0.02
Epilepsy	3120 (2.1)	1267 (1.5)	1727 (2.8)	0.08	2580.4 (1.8)	3248.4 (2.1)	0.02
Alcohol use disorder	12351 (8.3)	5916 (7.2)	5863 (9.4)	0.08	11121.2 (7.7)	12553.3 (8.1)	0.02
Substance use disorder	12909 (8.7)	5958 (7.3)	6329 (10.1)	0.10	11499.0 (7.9)	13277.4 (8.6)	0.02
Cardiovascular disease	5005 (3.4)	2162 (2.6)	2629 (4.2)	0.09	4221.9 (2.9)	5124.6 (3.3)	0.02
Type 2 diabetes	1094 (0.7)	445 (0.5)	603 (1.0)	0.05	865.1 (0.6)	1115.3 (0.7)	0.02
Dyslipidemia	570 (0.4)	251 (0.3)	299 (0.5)	0.03	486.4 (0.3)	593.6 (0.4)	0.01
Accidental Injuries	78481 (52.8)	42916 (52.5)	32430 (51.9)	0.01	75648.5 (52.2)	80916.7 (52.2)	<0.01
**Psychotropic medication use at baseline**							
Opioids [e]	30496 (20.5)	16288 (19.9)	13014 (20.8)	0.02	28728.7 (19.8)	31649.1 (20.4)	0.01
Antiepileptic drugs	10743 (7.2)	5021 (6.1)	5332 (8.5)	0.09	9532.8 (6.6)	11187.4 (7.2)	0.03
Antipsychotics	14842 (10.0)	7176 (8.8)	7105 (11.4)	0.09	13351.6 (9.2)	15288.7 (9.9)	0.02
Anxiolytics, hypnotics, and sedatives	56763 (38.2)	30158 (36.9)	24758 (39.6)	0.06	54221.8 (37.4)	59095.3 (38.1)	0.01
Antidepressants	51506 (34.7)	26921 (32.9)	23040 (36.9)	0.08	48827.3 (33.7)	53953.2 (34.8)	0.02
Anti-addiction drugs [f]	7549 (5.1)	3726 (4.6)	3479 (5.6)	0.05	6732.5 (4.6)	7814.0 (5.0)	0.02
Number of prior hospitalizations for psychiatric reason				0.14			0.03

0	124644 (83.9)	70501 (86.2)	50840 (81.4)		123445.0 (85.2)	130568.2 (84.2)	
1-2	15306 (10.3)	7545 (9.2)	7204 (11.5)		14139.2 (9.8)	15829.5 (10.2)	
3-4	3639 (2.4)	1656 (2.0)	1818 (2.9)		3164.5 (2.2)	3724.8 (2.4)	
5+	4992 (3.4)	2104 (2.6)	2609 (4.2)		4189.7 (2.9)	5012.2 (3.2)	
Number of prior outpatient visit for psychiatric reason				0.12			0.02
0	81542 (54.9)	46782 (57.2)	32555 (52.1)		81259.3 (56.1)	85745.7 (55.3)	
1-4	38872 (26.2)	20978 (25.6)	16719 (26.8)		37555.0 (25.9)	40270.4 (26.0)	
5-9	13639 (9.2)	6931 (8.5)	6280 (10.1)		12779.4 (8.8)	14068.8 (9.1)	
10+	14528 (9.8)	7115 (8.7)	6917 (11.1)		13344.6 (9.2)	15049.8 (9.7)	
Number of prior hospitalizations for non-psychiatric reason				0.12			0.03
0	79491 (53.5)	45464 (55.6)	32194 (51.5)		79504.1 (54.9)	83344.0 (53.7)	
1-2	48535 (32.7)	26489 (32.4)	20513 (32.8)		47063.3 (32.5)	50608.8 (32.6)	
3-4	11534 (7.8)	5866 (7.2)	5211 (8.3)		10705.5 (7.4)	11936.2 (7.7)	
5+	9021 (6.1)	3987 (4.9)	4553 (7.3)		7665.5 (5.3)	9245.7 (6.0)	
Number of prior outpatient visits for non-psychiatric reason				0.05			0.01
0	25989 (17.5)	14267 (17.4)	11331 (18.1)		25390.3 (17.5)	27373.9 (17.6)	
1-4	65142 (43.8)	36721 (44.9)	26871 (43.0)		64590.3 (44.6)	68305.8 (44.0)	
5-9	31635 (21.3)	17456 (21.3)	13039 (20.9)		30721.8 (21.2)	32905.4 (21.2)	
10+	25815 (17.4)	13362 (16.3)	11230 (18.0)		24235.9 (16.7)	26549.5 (17.1)	
**Time varying covariates in the previous month**							
Anxiety disorders	1129 (0.8)	618 (0.8)	516 (0.8)	0.01	1179.9 (0.8)	1201.1 (0.8)	<0.01
Autism spectrum disorder	2824 (1.9)	1335 (1.6)	1253 (2.0)	0.03	2460.2 (1.7)	2987.2 (1.9)	0.02
Bipolar disorder	1179 (0.8)	538 (0.7)	548 (0.9)	0.03	1021.9 (0.7)	1213.5 (0.8)	0.01
Conduct disorder	282 (0.2)	189 (0.2)	91 (0.1)	0.02	332.1 (0.2)	249.2 (0.2)	0.02
Depressive disorder	2433 (1.6)	1309 (1.6)	1053 (1.7)	0.01	2354.9 (1.6)	2611.5 (1.7)	<0.01
Eating disorder	253 (0.2)	124 (0.2)	132 (0.2)	0.01	228.8 (0.2)	292.0 (0.2)	0.01
Intellectual disability	381 (0.3)	180 (0.2)	178 (0.3)	0.01	353.5 (0.2)	365.9 (0.2)	<0.01
Personality disorder	910 (0.6)	403 (0.5)	452 (0.7)	0.03	799.5 (0.6)	978.5 (0.6)	0.01
Schizophrenia	319 (0.2)	112 (0.1)	175 (0.3)	0.03	236.3 (0.2)	312.1 (0.2)	0.01
Epilepsy	278 (0.2)	96 (0.1)	175 (0.3)	0.04	205.0 (0.1)	352.3 (0.2)	0.02
Alcohol use disorder	604 (0.4)	244 (0.3)	289 (0.5)	0.03	460.0 (0.3)	603.4 (0.4)	0.01
Substance use disorder	1265 (0.9)	541 (0.7)	653 (1.0)	0.04	1047.7 (0.7)	1404.1 (0.9)	0.02
Cardiovascular disease	350 (0.2)	148 (0.2)	157 (0.3)	0.02	282.2 (0.2)	319.9 (0.2)	<0.01
Type 2 diabetes	96 (0.1)	22 (0.0)	50 (0.1)	0.02	45.2 (0.0)	90.6 (0.1)	0.01
Dyslipidemia	18 (0.0)	9 (0.0)	11 (0.0)	0.01	17.9 (0.0)	22.6 (0.0)	<0.01
Accidental injuries	1462 (1.0)	776 (0.9)	610 (1.0)	<0.01	1379.0 (1.0)	1512.0 (1.0)	<0.01
Opioids [e]	3082 (2.1)	1547 (1.9)	1329 (2.1)	0.02	2782.5 (1.9)	3145.1 (2.0)	0.01
Antiepileptic drugs	3394 (2.3)	1483 (1.8)	1711 (2.7)	0.06	2850.2 (2.0)	3627.1 (2.3)	0.03
Antipsychotics	4769 (3.2)	2244 (2.7)	2248 (3.6)	0.05	4132.1 (2.9)	4823.3 (3.1)	0.02
Anxiolytics, hypnotics, and sedatives	19374 (13.0)	11211 (13.7)	7161 (11.5)	0.07	19151.8 (13.2)	18250.4 (11.8)	0.04
Antidepressants	13055 (8.8)	6961 (8.5)	5723 (9.2)	0.02	12557.2 (8.7)	13679.1 (8.8)	0.01
Anti-addiction drugs [f]	781 (0.5)	366 (0.4)	355 (0.6)	0.02	672.6 (0.5)	787.9 (0.5)	0.01
Any hospitalizations for psychiatric reasons	1403 (0.9)	551 (0.7)	656 (1.1)	0.04	1060.8 (0.7)	1411.9 (0.9)	0.02
Any outpatient visits for psychiatric reasons	19501 (13.1)	11546 (14.1)	7586 (12.1)	0.06	20537.1 (14.2)	18960.8 (12.2)	0.06
Any hospitalizations for non-psychiatric reasons	995 (0.7)	389 (0.5)	446 (0.7)	0.03	730.5 (0.5)	961.5 (0.6)	0.02
Any outpatient visits for non-psychiatric reasons	7333 (4.9)	3765 (4.6)	3147 (5.0)	0.02	6792.5 (4.7)	7553.5 (4.9)	0.01

SMD, standardized mean difference. Data are numbers (%) unless stated otherwise. [a] Assessed at baseline. [b] Assessed at baseline. Those who died or emigrated and did not initiate ADHD medication during the grace period (n = 78) contributed to both treatment strategies. [c] Including all countries other than Sweden. [d] For those younger than 25 years, education level was replaced by parents' highest education level. [e] Refers to prescribed opioids in the Prescription Drug Register. [f] Including drugs used in nicotine dependence, drugs used in alcohol dependence, and drugs used in opioid dependence.

Supplementary table H. Covariate balance at the end of grace period before and after weighting for transport accidents.

	Before weighting				After weighting		
Characteristics	Overall	Initiation	Non-initiation	SMD	Initiation	Non-initiation	SMD
N	89672	48251	40582		89820.3	95109.6	
**Age at baseline (median, IQR)**	26.1 [18.7, 36.6]	25.4 [17.9, 36.2]	26.7 [20.0, 37.2]	0.11	25.4 [17.9, 36.1]	26.0 [19.6, 36.2]	0.07
**Sex**							
Male	44982 (50.2)	23728 (49.2)	20672 (50.9)	0.04	44150.1 (49.2)	47185.0 (49.6)	0.01
Female	44690 (49.8)	24523 (50.8)	19910 (49.1)	0.04	45670.2 (50.8)	47924.6 (50.4)	0.01
**Calendar year at baseline**				0.13			0.04
2007	2955 (3.3)	1256 (2.6)	1652 (4.1)		2656.6 (3.0)	3121.3 (3.3)	
2008	4266 (4.8)	1891 (3.9)	2314 (5.7)		3871.4 (4.3)	4571.9 (4.8)	
2009	5132 (5.7)	2554 (5.3)	2512 (6.2)		4850.0 (5.4)	5480.9 (5.8)	
2010	6425 (7.2)	3450 (7.2)	2905 (7.2)		6300.9 (7.0)	6933.7 (7.3)	
2011	7382 (8.2)	4131 (8.6)	3171 (7.8)		7381.7 (8.2)	7731.7 (8.1)	
2012	7746 (8.6)	4183 (8.7)	3492 (8.6)		7656.0 (8.5)	8284.3 (8.7)	
2013	8002 (8.9)	4303 (8.9)	3629 (8.9)		8073.6 (9.0)	8525.0 (9.0)	
2014	8808 (9.8)	4853 (10.1)	3877 (9.6)		9095.6 (10.1)	9261.3 (9.7)	
2015	9316 (10.4)	5254 (10.9)	3989 (9.8)		9750.5 (10.9)	9748.0 (10.2)	
2016	9712 (10.8)	5277 (10.9)	4356 (10.7)		9923.1 (11.0)	10385.1 (10.9)	
2017	9859 (11.0)	5389 (11.2)	4387 (10.8)		9992.0 (11.1)	10482.6 (11.0)	
2018	10069 (11.2)	5710 (11.8)	4298 (10.6)		10268.9 (11.4)	10583.7 (11.1)	
**Birth country**							
Sweden	81628 (91.0)	44207 (91.6)	36652 (90.3)	0.05	82036.9 (91.3)	86695.5 (91.2)	0.01
Other[c]	8044 (9.0)	4044 (8.4)	3930 (9.7)	0.05	7783.4 (8.7)	8414.1 (8.8)	0.01
**Education level at baseline[d]**				0.15			0.04
Primary or lower secondary	17159 (19.1)	8155 (16.9)	8783 (21.6)		16158.3 (18.0)	18342.1 (19.3)	
Upper secondary	46405 (51.7)	24903 (51.6)	21061 (51.9)		46321.6 (51.6)	49228.6 (51.8)	
Post-secondary or postgraduate	25288 (28.2)	14842 (30.8)	10296 (25.4)		26630.7 (29.6)	26731.4 (28.1)	
Unknown	820 (0.9)	351 (0.7)	442 (1.1)		709.7 (0.8)	807.6 (0.8)	
**Comorbidities at baseline**							
Anxiety disorders	11672 (13.0)	5813 (12.0)	5746 (14.2)	0.06	11244.0 (12.5)	12485.5 (13.1)	0.02
Autism spectrum disorder	2925 (3.3)	1253 (2.6)	1656 (4.1)	0.08	2566.4 (2.9)	2972.3 (3.1)	0.02
Bipolar disorder	5101 (5.7)	2508 (5.2)	2551 (6.3)	0.05	4770.4 (5.3)	5514.6 (5.8)	0.02
Conduct disorder	1082 (1.2)	542 (1.1)	525 (1.3)	0.02	1081.2 (1.2)	1120.8 (1.2)	<0.01
Depressive disorder	27192 (30.3)	14255 (29.5)	12717 (31.3)	0.04	26844.7 (29.9)	29562.3 (31.1)	0.03
Eating disorder	3078 (3.4)	1626 (3.4)	1437 (3.5)	0.01	3107.0 (3.5)	3273.8 (3.4)	<0.01
Intellectual disability	1272 (1.4)	435 (0.9)	828 (2.0)	0.09	974.0 (1.1)	1295.4 (1.4)	0.03
Personality disorder	7230 (8.1)	3374 (7.0)	3789 (9.3)	0.09	6753.9 (7.5)	7779.2 (8.2)	0.02
Schizophrenia	2562 (2.9)	1033 (2.1)	1495 (3.7)	0.09	2197.6 (2.4)	2663.1 (2.8)	0.02
Epilepsy	1873 (2.1)	769 (1.6)	1073 (2.6)	0.07	1639.0 (1.8)	1964.1 (2.1)	0.02
Alcohol use disorder	12238 (13.6)	6001 (12.4)	6027 (14.9)	0.07	11602.3 (12.9)	12970.1 (13.6)	0.02
Substance use disorder	12782 (14.3)	6033 (12.5)	6473 (16.0)	0.10	11996.3 (13.4)	13678.5 (14.4)	0.03
Cardiovascular disease	4492 (5.0)	2007 (4.2)	2424 (6.0)	0.08	4020.3 (4.5)	4742.1 (5.0)	0.02
Type 2 diabetes	1076 (1.2)	443 (0.9)	624 (1.5)	0.06	888.7 (1.0)	1172.3 (1.2)	0.02
Dyslipidemia	543 (0.6)	232 (0.5)	306 (0.8)	0.03	473.2 (0.5)	599.7 (0.6)	0.01
Transport accidents	16720 (18.6)	8245 (17.1)	8061 (19.9)	0.07	15599.6 (17.4)	17653.6 (18.6)	0.03
**Psychotropic medication use at baseline**							
Opioids [e]	28886 (32.2)	15750 (32.6)	12786 (31.5)	0.02	28468.9 (31.7)	31042.6 (32.6)	0.02
Antiepileptic drugs	9526 (10.6)	4623 (9.6)	4795 (11.8)	0.07	8964.1 (10.0)	10287.6 (10.8)	0.03
Antipsychotics	14245 (15.9)	6953 (14.4)	7120 (17.5)	0.09	13435.3 (15.0)	15288.6 (16.1)	0.03
Anxiolytics, hypnotics, and sedatives	50200 (56.0)	26700 (55.3)	23008 (56.7)	0.03	49618.4 (55.2)	54053.7 (56.8)	0.03
Antidepressants	49563 (55.3)	26232 (54.4)	22906 (56.4)	0.04	48872.2 (54.4)	53616.6 (56.4)	0.04
Anti-addiction drugs [f]	7546 (8.4)	3833 (7.9)	3604 (8.9)	0.03	7085.5 (7.9)	8159.5 (8.6)	0.03
Number of prior hospitalizations for psychiatric reason				0.14			0.04

0	66589 (74.3)	37127 (76.9)	28964 (71.4)		68032.0 (75.7)	70452.0 (74.1)	
1-2	14556 (16.2)	7288 (15.1)	7071 (17.4)		14048.9 (15.6)	15571.4 (16.4)	
3-4	3573 (4.0)	1678 (3.5)	1828 (4.5)		3289.1 (3.7)	3793.4 (4.0)	
5+	4954 (5.5)	2158 (4.5)	2719 (6.7)		4450.4 (5.0)	5292.9 (5.6)	
Number of prior outpatient visit for psychiatric reason				0.11			0.03
0	36454 (40.7)	20754 (43.0)	15388 (37.9)		37524.0 (41.8)	38279.0 (40.2)	
1-4	27153 (30.3)	14319 (29.7)	12551 (30.9)		27053.8 (30.1)	28937.8 (30.4)	
5-9	12071 (13.5)	6182 (12.8)	5763 (14.2)		11717.6 (13.0)	12851.8 (13.5)	
10+	13994 (15.6)	6996 (14.5)	6880 (17.0)		13524.9 (15.1)	15041.1 (15.8)	
Number of prior hospitalizations for non-psychiatric reason				0.12			0.05
0	39139 (43.6)	21972 (45.5)	16922 (41.7)		40500.5 (45.1)	41219.7 (43.3)	
1-2	33170 (37.0)	17856 (37.0)	14988 (36.9)		33051.7 (36.8)	35216.4 (37.0)	
3-4	9463 (10.6)	4828 (10.0)	4498 (11.1)		9130.4 (10.2)	10160.6 (10.7)	
5+	7900 (8.8)	3595 (7.5)	4174 (10.3)		7137.7 (7.9)	8513.0 (9.0)	
Number of prior outpatient visits for non-psychiatric reason				0.03			0.01
0	14322 (16.0)	7604 (15.8)	6627 (16.3)		14213.7 (15.8)	15103.7 (15.9)	
1-4	37512 (41.8)	20407 (42.3)	16793 (41.4)		37938.6 (42.2)	39679.9 (41.7)	
5-9	20104 (22.4)	10941 (22.7)	8932 (22.0)		20100.1 (22.4)	21359.4 (22.5)	
10+	17734 (19.8)	9299 (19.3)	8230 (20.3)		17567.9 (19.6)	18966.7 (19.9)	
**Time varying covariates in the previous month**							
Anxiety disorders	1011 (1.1)	541 (1.1)	485 (1.2)	0.01	1070.8 (1.2)	1115.5 (1.2)	<0.01
Autism spectrum disorder	1652 (1.8)	732 (1.5)	818 (2.0)	0.04	1436.9 (1.6)	1813.8 (1.9)	0.02
Bipolar disorder	1131 (1.3)	521 (1.1)	554 (1.4)	0.03	1025.1 (1.1)	1237.2 (1.3)	0.01
Conduct disorder	40 (0.0)	19 (0.0)	15 (0.0)	<0.01	40.0 (0.0)	33.8 (0.0)	<0.01
Depressive disorder	2158 (2.4)	1181 (2.4)	984 (2.4)	<0.01	2201.1 (2.5)	2388.2 (2.5)	<0.01
Eating disorder	215 (0.2)	113 (0.2)	120 (0.3)	0.01	218.9 (0.2)	267.6 (0.3)	0.01
Intellectual disability	195 (0.2)	77 (0.2)	101 (0.2)	0.02	163.6 (0.2)	190.3 (0.2)	<0.01
Personality disorder	906 (1.0)	416 (0.9)	471 (1.2)	0.03	851.9 (0.9)	1025.4 (1.1)	0.01
Schizophrenia	311 (0.3)	117 (0.2)	185 (0.5)	0.04	252.1 (0.3)	331.9 (0.3)	0.01
Epilepsy	116 (0.1)	41 (0.1)	72 (0.2)	0.03	89.1 (0.1)	132.1 (0.1)	0.01
Alcohol use disorder	588 (0.7)	245 (0.5)	294 (0.7)	0.03	483.5 (0.5)	618.8 (0.7)	0.01
Substance use disorder	1227 (1.4)	543 (1.1)	668 (1.6)	0.04	1095.0 (1.2)	1421.9 (1.5)	0.02
Cardiovascular disease	309 (0.3)	133 (0.3)	146 (0.4)	0.01	260.7 (0.3)	300.2 (0.3)	<0.01
Type 2 diabetes	92 (0.1)	22 (0.0)	50 (0.1)	0.03	45.5 (0.1)	89.2 (0.1)	0.02
Dyslipidemia	15 (0.0)	6 (0.0)	10 (0.0)	0.01	12.6 (0.0)	19.7 (0.0)	0.01
Transport accidents	246 (0.3)	112 (0.2)	108 (0.3)	0.01	212.5 (0.2)	238.3 (0.3)	<0.01
Opioids [e]	3040 (3.4)	1579 (3.3)	1350 (3.3)	<0.01	2894.9 (3.2)	3225.3 (3.4)	0.01
Antiepileptic drugs	2795 (3.1)	1282 (2.7)	1435 (3.5)	0.05	2525.2 (2.8)	3060.7 (3.2)	0.02
Antipsychotics	4145 (4.6)	1859 (3.9)	2134 (5.3)	0.07	3672.9 (4.1)	4454.2 (4.7)	0.03
Anxiolytics, hypnotics, and sedatives	13539 (15.1)	7472 (15.5)	5768 (14.2)	0.04	13580.3 (15.1)	13773.0 (14.5)	0.02
Antidepressants	11495 (12.8)	6155 (12.8)	5302 (13.1)	0.01	11501.4 (12.8)	12457.2 (13.1)	0.01
Anti-addiction drugs [f]	778 (0.9)	382 (0.8)	372 (0.9)	0.01	721.6 (0.8)	820.9 (0.9)	0.01
Any hospitalizations for psychiatric reasons	1294 (1.4)	513 (1.1)	648 (1.6)	0.05	1034.3 (1.2)	1389.2 (1.5)	0.03
Any outpatient visits for psychiatric reasons	12476 (13.9)	7191 (14.9)	5415 (13.3)	0.04	13454.6 (15.0)	12844.8 (13.5)	0.04
Any hospitalizations for non-psychiatric reasons	865 (1.0)	328 (0.7)	405 (1.0)	0.03	646.8 (0.7)	867.1 (0.9)	0.02
Any outpatient visits for non-psychiatric reasons	4889 (5.5)	2490 (5.2)	2269 (5.6)	0.02	4698.6 (5.2)	5248.7 (5.5)	0.01

SMD, standardized mean difference. Data are numbers (%) unless stated otherwise. [a] Assessed at baseline. [b] Assessed at baseline. Those who died or emigrated and did not initiate ADHD medication during the grace period (n = 78) contributed to both treatment strategies. [c] Including all countries other than Sweden. [d] For those younger than 25 years, education level was replaced by parents' highest education level. [e] Refers to prescribed opioids in the Prescription Drug Register. [f] Including drugs used in nicotine dependence, drugs used in alcohol dependence, and drugs used in opioid dependence.

Supplementary table I. Covariate balance at the end of grace period before and after weighting for criminality.

		Before weighting			After weighting		
Characteristics	Overall	Initiation	Non-initiation	SMD	Initiation	Non-initiation	SMD
N	89672	47460	39511		87488.9	92711.1	
**Age at baseline (median, IQR)**	26.1 [18.7, 36.6]	25.4 [17.9, 36.2]	26.8 [20.0, 37.2]	0.12	25.4 [17.9, 36.1]	26.1 [19.6, 36.3]	0.07
**Sex**							
Male	44982 (50.2)	23108 (48.7)	19792 (50.1)	0.03	42374.7 (48.4)	45262.2 (48.8)	0.01
Female	44690 (49.8)	24352 (51.3)	19719 (49.9)	0.03	45114.2 (51.6)	47448.9 (51.2)	0.01
**Calendar year at baseline**				0.13			0.04
2007	2955 (3.3)	1208 (2.5)	1574 (4.0)		2512.5 (2.9)	2953.1 (3.2)	
2008	4266 (4.8)	1830 (3.9)	2197 (5.6)		3684.8 (4.2)	4319.5 (4.7)	
2009	5132 (5.7)	2471 (5.2)	2399 (6.1)		4644.8 (5.3)	5198.4 (5.6)	
2010	6425 (7.2)	3337 (7.0)	2781 (7.0)		6003.4 (6.9)	6621.8 (7.1)	
2011	7382 (8.2)	4020 (8.5)	3094 (7.8)		7132.7 (8.2)	7543.9 (8.1)	
2012	7746 (8.6)	4099 (8.6)	3381 (8.6)		7435.2 (8.5)	8042.7 (8.7)	
2013	8002 (8.9)	4241 (8.9)	3544 (9.0)		7871.2 (9.0)	8325.4 (9.0)	
2014	8808 (9.8)	4795 (10.1)	3785 (9.6)		8891.8 (10.2)	9054.1 (9.8)	
2015	9316 (10.4)	5211 (11.0)	3911 (9.9)		9586.5 (11.0)	9604.4 (10.4)	
2016	9712 (10.8)	5227 (11.0)	4295 (10.9)		9747.9 (11.1)	10265.5 (11.1)	
2017	9859 (11.0)	5358 (11.3)	4314 (10.9)		9846.4 (11.3)	10326.9 (11.1)	
2018	10069 (11.2)	5663 (11.9)	4236 (10.7)		10131.7 (11.6)	10455.4 (11.3)	
**Birth country**							
Sweden	81628 (91.0)	43504 (91.7)	35729 (90.4)	0.04	79961.2 (91.4)	84563.9 (91.2)	0.01
Other[c]	8044 (9.0)	3956 (8.3)	3782 (9.6)	0.04	7527.7 (8.6)	8147.2 (8.8)	0.01
**Education level at baseline[d]**				0.15			0.04
Primary or lower secondary	17159 (19.1)	7886 (16.6)	8379 (21.2)		15412.6 (17.6)	17485.6 (18.9)	
Upper secondary	46405 (51.7)	24504 (51.6)	20548 (52.0)		45142.3 (51.6)	48024.5 (51.8)	
Post-secondary or postgraduate	25288 (28.2)	14732 (31.0)	10159 (25.7)		26261.8 (30.0)	26419.3 (28.5)	
Unknown	820 (0.9)	338 (0.7)	425 (1.1)		672.2 (0.8)	781.7 (0.8)	
**Comorbidities at baseline**							
Anxiety disorders	11672 (13.0)	5746 (12.1)	5662 (14.3)	0.07	11052.7 (12.6)	12287.3 (13.3)	0.02
Autism spectrum disorder	2925 (3.3)	1236 (2.6)	1625 (4.1)	0.08	2511.1 (2.9)	2928.2 (3.2)	0.02
Bipolar disorder	5101 (5.7)	2478 (5.2)	2515 (6.4)	0.05	4697.8 (5.4)	5456.3 (5.9)	0.02
Conduct disorder	1082 (1.2)	521 (1.1)	490 (1.2)	0.01	1027.8 (1.2)	1046.3 (1.1)	<0.01
Depressive disorder	27192 (30.3)	14082 (29.7)	12479 (31.6)	0.04	26300.9 (30.1)	29017.9 (31.3)	0.03
Eating disorder	3078 (3.4)	1615 (3.4)	1426 (3.6)	0.01	3069.6 (3.5)	3244.4 (3.5)	<0.01
Intellectual disability	1272 (1.4)	421 (0.9)	804 (2.0)	0.10	936.6 (1.1)	1253.9 (1.4)	0.03
Personality disorder	7230 (8.1)	3307 (7.0)	3670 (9.3)	0.08	6565.6 (7.5)	7547.9 (8.1)	0.02
Schizophrenia	2562 (2.9)	995 (2.1)	1430 (3.6)	0.09	2088.1 (2.4)	2530.6 (2.7)	0.02
Epilepsy	1873 (2.1)	745 (1.6)	1040 (2.6)	0.07	1574.8 (1.8)	1900.6 (2.0)	0.02
Alcohol use disorder	12238 (13.6)	5802 (12.2)	5710 (14.5)	0.07	10995.6 (12.6)	12287.5 (13.3)	0.02
Substance use disorder	12782 (14.3)	5733 (12.1)	5931 (15.0)	0.09	11082.2 (12.7)	12526.1 (13.5)	0.03
Cardiovascular disease	4492 (5.0)	1971 (4.2)	2378 (6.0)	0.08	3921.4 (4.5)	4653.0 (5.0)	0.03
Type 2 diabetes	1076 (1.2)	435 (0.9)	606 (1.5)	0.06	862.5 (1.0)	1131.6 (1.2)	0.02
Dyslipidemia	543 (0.6)	229 (0.5)	302 (0.8)	0.04	466.0 (0.5)	585.6 (0.6)	0.01
Criminality	33182 (37.0)	15709 (33.1)	15351 (38.9)	0.12	29592.5 (33.8)	33442.8 (36.1)	0.05
**Psychotropic medication use at baseline**							
Opioids [e]	28886 (32.2)	15496 (32.7)	12459 (31.5)	0.02	27736.6 (31.7)	30279.7 (32.7)	0.02
Antiepileptic drugs	9526 (10.6)	4525 (9.5)	4644 (11.8)	0.07	8676.5 (9.9)	9969.7 (10.8)	0.03
Antipsychotics	14245 (15.9)	6786 (14.3)	6871 (17.4)	0.08	12946.6 (14.8)	14716.8 (15.9)	0.03
Anxiolytics, hypnotics, and sedatives	50200 (56.0)	26281 (55.4)	22429 (56.8)	0.03	48373.3 (55.3)	52779.8 (56.9)	0.03
Antidepressants	49563 (55.3)	25839 (54.4)	22411 (56.7)	0.05	47750.3 (54.6)	52509.7 (56.6)	0.04
Anti-addiction drugs [f]	7546 (8.4)	3719 (7.8)	3418 (8.7)	0.03	6745.5 (7.7)	7756.3 (8.4)	0.02
Number of prior hospitalizations for psychiatric reason				0.13			0.04

0	66589 (74.3)	36652 (77.2)	28427 (71.9)		66655.3 (76.2)	69186.7 (74.6)	
1-2	14556 (16.2)	7117 (15.0)	6812 (17.2)		13543.1 (15.5)	15010.8 (16.2)	
3-4	3573 (4.0)	1626 (3.4)	1737 (4.4)		3130.5 (3.6)	3599.3 (3.9)	
5+	4954 (5.5)	2065 (4.4)	2535 (6.4)		4160.0 (4.8)	4914.3 (5.3)	
Number of prior outpatient visit for psychiatric reason				0.11			0.03
0	36454 (40.7)	20463 (43.1)	15047 (38.1)		36692.8 (41.9)	37486.5 (40.4)	
1-4	27153 (30.3)	14040 (29.6)	12157 (30.8)		26225.2 (30.0)	28054.5 (30.3)	
5-9	12071 (13.5)	6069 (12.8)	5603 (14.2)		11383.1 (13.0)	12511.6 (13.5)	
10+	13994 (15.6)	6888 (14.5)	6704 (17.0)		13187.9 (15.1)	14658.5 (15.8)	
Number of prior hospitalizations for non-psychiatric reason				0.11			0.04
0	39139 (43.6)	21663 (45.6)	16606 (42.0)		39622.7 (45.3)	40485.8 (43.7)	
1-2	33170 (37.0)	17595 (37.1)	14562 (36.9)		32217.0 (36.8)	34290.8 (37.0)	
3-4	9463 (10.6)	4722 (9.9)	4341 (11.0)		8821.7 (10.1)	9812.6 (10.6)	
5+	7900 (8.8)	3480 (7.3)	4002 (10.1)		6827.6 (7.8)	8122.0 (8.8)	
Number of prior outpatient visits for non-psychiatric reason				0.03			0.01
0	14322 (16.0)	7487 (15.8)	6491 (16.4)		13870.8 (15.9)	14803.9 (16.0)	
1-4	37512 (41.8)	20044 (42.2)	16343 (41.4)		36955.3 (42.2)	38675.5 (41.7)	
5-9	20104 (22.4)	10758 (22.7)	8661 (21.9)		19551.7 (22.3)	20755.5 (22.4)	
10+	17734 (19.8)	9171 (19.3)	8016 (20.3)		17111.1 (19.6)	18476.2 (19.9)	
**Time varying covariates in the previous month**							
Anxiety disorders	1011 (1.1)	536 (1.1)	480 (1.2)	0.01	1058.2 (1.2)	1100.0 (1.2)	<0.01
Autism spectrum disorder	1652 (1.8)	723 (1.5)	809 (2.0)	0.04	1407.1 (1.6)	1793.6 (1.9)	0.02
Bipolar disorder	1131 (1.3)	513 (1.1)	542 (1.4)	0.03	1000.9 (1.1)	1208.9 (1.3)	0.01
Conduct disorder	40 (0.0)	16 (0.0)	14 (0.0)	<0.01	31.9 (0.0)	30.8 (0.0)	<0.01
Depressive disorder	2158 (2.4)	1175 (2.5)	968 (2.4)	<0.01	2177.6 (2.5)	2358.3 (2.5)	<0.01
Eating disorder	215 (0.2)	113 (0.2)	118 (0.3)	0.01	218.1 (0.2)	259.2 (0.3)	0.01
Intellectual disability	195 (0.2)	74 (0.2)	100 (0.3)	0.02	157.7 (0.2)	187.2 (0.2)	<0.01
Personality disorder	906 (1.0)	412 (0.9)	460 (1.2)	0.03	838.4 (1.0)	998.2 (1.1)	0.01
Schizophrenia	311 (0.3)	108 (0.2)	174 (0.4)	0.04	229.4 (0.3)	311.6 (0.3)	0.01
Epilepsy	116 (0.1)	40 (0.1)	72 (0.2)	0.03	85.5 (0.1)	130.1 (0.1)	0.01
Alcohol use disorder	588 (0.7)	231 (0.5)	268 (0.7)	0.03	448.7 (0.5)	560.1 (0.6)	0.01
Substance use disorder	1227 (1.4)	506 (1.1)	579 (1.5)	0.04	970.9 (1.1)	1221.2 (1.3)	0.02
Cardiovascular disease	309 (0.3)	133 (0.3)	142 (0.4)	0.01	256.3 (0.3)	293.5 (0.3)	<0.01
Type 2 diabetes	92 (0.1)	20 (0.0)	49 (0.1)	0.03	40.3 (0.0)	87.2 (0.1)	0.02
Dyslipidemia	15 (0.0)	6 (0.0)	10 (0.0)	0.01	12.3 (0.0)	19.9 (0.0)	0.01
Criminality	882 (1.0)	350 (0.7)	356 (0.9)	0.02	681.0 (0.8)	772.0 (0.8)	0.01
Opioids [e]	3040 (3.4)	1551 (3.3)	1321 (3.3)	<0.01	2819.2 (3.2)	3141.4 (3.4)	0.01
Antiepileptic drugs	2795 (3.1)	1263 (2.7)	1409 (3.6)	0.05	2471.1 (2.8)	3002.3 (3.2)	0.02
Antipsychotics	4145 (4.6)	1815 (3.8)	2063 (5.2)	0.07	3553.6 (4.1)	4290.1 (4.6)	0.03
Anxiolytics, hypnotics, and sedatives	13539 (15.1)	7333 (15.5)	5624 (14.2)	0.03	13191.2 (15.1)	13417.3 (14.5)	0.02
Antidepressants	11495 (12.8)	6087 (12.8)	5210 (13.2)	0.01	11282.2 (12.9)	12261.9 (13.2)	0.01
Anti-addiction drugs [f]	778 (0.9)	366 (0.8)	342 (0.9)	0.01	670.7 (0.8)	750.5 (0.8)	<0.01
Any hospitalizations for psychiatric reasons	1294 (1.4)	487 (1.0)	601 (1.5)	0.04	961.9 (1.1)	1278.4 (1.4)	0.03
Any outpatient visits for psychiatric reasons	12476 (13.9)	7083 (14.9)	5269 (13.3)	0.05	13119.0 (15.0)	12509.3 (13.5)	0.04
Any hospitalizations for non-psychiatric reasons	865 (1.0)	318 (0.7)	383 (1.0)	0.03	616.5 (0.7)	819.2 (0.9)	0.02
Any outpatient visits for non-psychiatric reasons	4889 (5.5)	2449 (5.2)	2212 (5.6)	0.02	4579.5 (5.2)	5110.6 (5.5)	0.01

SMD, standardized mean difference. Data are numbers (%) unless stated otherwise. [a] Assessed at baseline. [b] Assessed at baseline. Those who died or emigrated and did not initiate ADHD medication during the grace period (n = 78) contributed to both treatment strategies. [c] Including all countries other than Sweden. [d] For those younger than 25 years, education level was replaced by parents' highest education level. [e] Refers to prescribed opioids in the Prescription Drug Register. [f] Including drugs used in nicotine dependence, drugs used in alcohol dependence, and drugs used in opioid dependence.

Supplementary table J. ADHD medication and first and recurrent event rates over 2 years of follow-up among individuals with ADHD, stimulants vs non-stimulants

	Stimulants			Non-stimulants			Incidence rate ratio (95% CI)
	Events	Person years	Incidence rate	Events	Person years	Incidence rate	
**First event**							
**Suicidal behaviours**	2,052	166,788	12.3	2,250	166,380	13.5	0.91 (0.85, 0.96)
**Substance misuse**	7,015	158,674	44.2	9,271	155,861	59.5	0.74 (0.72, 0.76)
**Accidental injuries**	13,122	153,722	85.4	13,690	153,463	89.2	0.95 (0.93, 0.98)
**Transport accidents**	2,059	95,318	21.6	2,465	95,143	25.9	0.83 (0.78, 0.88)
**Criminality**	5,011	91,266	54.9	6,316	89,642	70.5	0.78 (0.75, 0.81)
**Recurrent events**							
**Suicidal behaviours**	3,159	169,240	18.7	3,911	168,963	23.1	0.80 (0.76, 0.84)
**Substance misuse**	23,474	169,240	138.7	31,490	168,991	186.3	0.75 (0.73, 0.76)
**Accidental injuries**	19,982	169,239	118.1	20,778	168,994	123.0	0.97 (0.95, 0.99)
**Transport accidents**	2,837	97,595	29.1	3,608	97,731	36.9	0.78 (0.75, 0.82)
**Criminality**	9,487	97,595	97.2	13,177	97,762	134.8	0.71 (0.69, 0.73)

Incidence rates were calculated per 1,000 person-years. The numbers reported are weighted and account for follow-up censoring, including treatment discontinuation or switching. In the head-to-head comparison, those who have events, migrated, or died before medication initiation are not included in the analysis.

Supplementary table K. ADHD medication and rates of first event over 2 years of follow-up among individuals with ADHD, by age groups.

	Initiation			Non-initiation			Incidence rate ratio (95% CI)
	Events	Person years	Incidence rate	Events	Person years	Incidence rate	
**6-24 years**							
**Suicidal behaviours**	2,550	207,355	12.3	2,885	203,713	14.2	0.84 (0.77, 0.91)
**Substance misuse**	7,594	200,698	37.8	7,997	196,440	40.7	0.92 (0.88, 0.96)
**Accidental injuries**	16,990	190,159	89.3	16,440	188,011	87.4	1.02 (0.98, 1.06)
**Transport accidents**	2,012	85,556	23.5	2,184	83,719	26.1	0.91 (0.81, 1.01)
**Criminality**	5,760	80,493	71.6	6,276	78,243	80.2	0.90 (0.85, 0.95)
**25-64 years**							
**Suicidal behaviours**	1,911	97,864	19.5	2,138	95,707	22.3	0.86 (0.78, 0.94)
**Substance misuse**	9,418	85,708	109.9	11,020	81,957	134.5	0.83 (0.80, 0.86)
**Accidental injuries**	7,957	90,731	87.7	8,446	88,721	95.2	0.93 (0.89, 0.98)
**Transport accidents**	2,362	97,265	24.3	2,719	94,983	28.6	0.86 (0.79, 0.99)
**Criminality**	5,432	92,933	58.4	6,595	89,903	73.4	0.81 (0.77, 0.85)

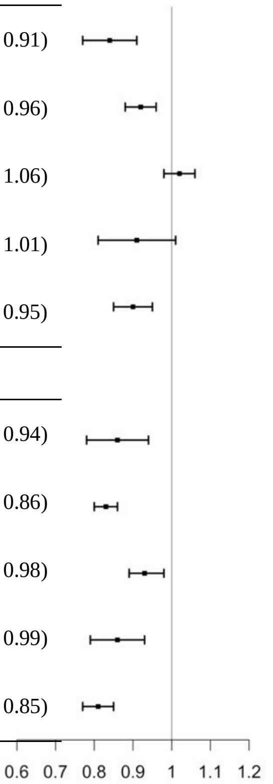

Incidence rates were calculated per 1,000 person-years. The numbers reported are weighted and account for follow-up censoring, including treatment discontinuation or switching. Wald test for differences between the IRRs for children and youths vs adults across the five outcomes, i.e. suicidal behaviours, substance misuse, unintentional injuries, transport accidents and criminality, yielded the following p-values: 0.71, <0.01, <0.01, 0.42, and <0.01, respectively.

Supplementary table L. ADHD medication and rates of first event over 2 years of follow-up among individuals with ADHD, by sex.

	Initiation			Non-initiation			Incidence rate ratio (95% CI)
	Events	Person years	Incidence rate	Events	Person years	Incidence rate	
**Male**							
**Suicidal behaviours**	1,895	180,096	10.5	2,245	176,503	12.7	0.81 (0.74, 0.89)
**Substance misuse**	10,439	167,123	62.5	12,016	161,922	74.2	0.84 (0.81, 0.86)
**Accidental injuries**	15,516	163,664	94.8	15,366	161,361	95.2	1.00 (0.97, 1.04)
**Transport accidents**	2,829	90,769	31.2	3,167	88,443	35.8	0.88 (0.81, 0.95)
**Criminality**	8,535	82,949	102.9	9,309	80,204	116.1	0.90 (0.86, 0.94)
**Female**							
**Suicidal behaviours**	2,525	125,268	20.2	2,824	123,044	23.0	0.86 (0.79, 0.94)
**Substance misuse**	6,401	119,551	53.5	7,122	116,511	61.1	0.87 (0.83, 0.91)
**Accidental injuries**	9,399	117,333	80.1	9,534	115,411	82.6	0.97 (0.92, 1.02)
**Transport accidents**	1,568	91,917	17.1	1,721	90,227	19.1	0.90 (0.78, 1.00)
**Criminality**	2,794	90,209	31.0	3,392	88,076	38.5	0.81 (0.74, 0.87)

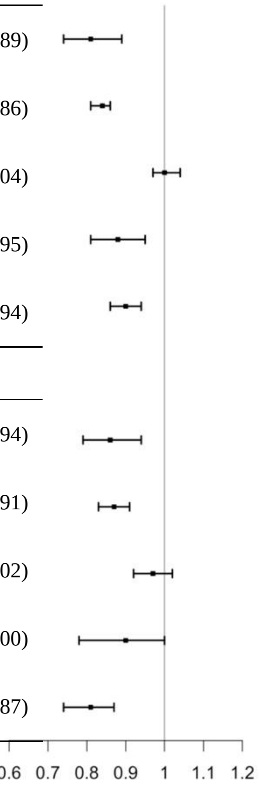

Incidence rates were calculated per 1,000 person-years. The numbers reported are weighted and account for follow-up censoring, including treatment discontinuation or switching. Wald test for differences between the IRRs for males versus females across the five outcomes, i.e. suicidal behaviours, substance misuse, unintentional injuries, transport accidents and criminality, yielded the following p-values: 0.35, 0.21, 0.34, 0.76 and 0.02, respectively.

Supplementary table M. ADHD medication and rates of recurrent events over 2 years of follow-up among individuals with ADHD, by age groups.

	Initiation			Non-initiation			Incidence rate ratio (95% CI)
	Events	Person years	Incidence rate	Events	Person years	Incidence rate	
**6-24 years**							
**Suicidal behaviours**	3,854	210,101	18.3	4,263	208,938	20.4	0.80 (0.70, 0.91)
**Substance misuse**	16,776	210,099	79.8	20,433	208,975	97.8	0.73 (0.68, 0.78)
**Accidental injuries**	24,760	210,103	117.8	24,406	208,978	116.8	1.00 (0.95, 1.04)
**Transport accidents**	2,499	87,642	28.5	2,831	86,294	32.8	0.88 (0.78, 0.98)
**Criminality**	10,020	87,646	114.3	12,049	86,308	139.6	0.80 (0.75, 0.86)
**25-64 years**							
**Suicidal behaviours**	3,235	100,218	32.3	3,126	98,485	31.7	0.96 (0.80, 1.10)
**Substance misuse**	35,304	100,216	352.3	40,959	98,487	415.9	0.80 (0.76, 0.84)
**Accidental injuries**	12,485	100,220	124.6	13,197	98,484	134.0	0.92 (0.87, 0.98)
**Transport accidents**	3,414	100,221	34.1	4,053	98,480	41.2	0.81 (0.71, 0.90)
**Criminality**	10,829	100,228	108.0	14,476	98,493	147.0	0.71 (0.66, 0.76)

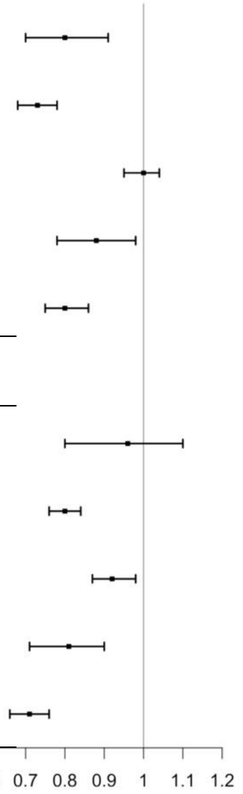

Incidence rates were calculated per 1,000 person-years. The numbers reported are weighted and account for follow-up censoring, including treatment discontinuation or switching. Wald test for differences between the IRRs for children and youths vs adults across the five outcomes, i.e. suicidal behaviours, substance misuse, unintentional injuries, transport accidents and criminality, yielded the following p-values: 0.08, 0.03, 0.03, 0.32, and 0.02, respectively.

Supplementary table N. ADHD medication and rates of recurrent events over 2 years of follow-up among individuals with ADHD, by sex.

	Initiation			Non-initiation			Incidence rate ratio (95% CI)
	Events	Person years	Event rate	Events	Person years	Event rate	
**Male**							
**Suicidal behaviours**	2,620	181,977	14.4	2,865	180,294	15.9	0.83 (0.72, 0.95)
**Substance misuse**	34,110	182,005	187.4	41,158	180,318	228.3	0.73 (0.69, 0.77)
**Accidental injuries**	23,295	181,980	128.0	23,324	180,302	129.4	0.98 (0.93, 1.02)
**Transport accidents**	4,034	93,843	43.0	4,620	92,391	50.0	0.85 (0.76, 0.94)
**Criminality**	16,605	93,860	176.9	20,335	92,416	220.0	0.78 (0.73, 0.83)
**Female**							
**Suicidal behaviours**	4,389	128,210	34.2	4,650	127,109	36.6	0.85 (0.73, 0.96)
**Substance misuse**	17,575	128,206	137.1	20,351	127,115	160.1	0.79 (0.73, 0.84)
**Accidental injuries**	13,803	128,210	107.7	14,379	127,110	113.1	0.94 (0.88, 1.00)
**Transport accidents**	1,927	93,500	20.6	2,230	92,372	24.1	0.85 (0.73, 0.97)
**Criminality**	4,482	93,500	47.9	5,892	92,374	63.8	0.73 (0.66, 0.80)

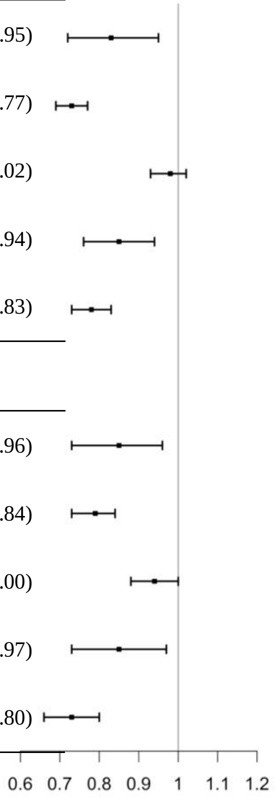

Incidence rates were calculated per 1,000 person-years. The numbers reported are weighted and account for follow-up censoring, including treatment discontinuation or switching. Wald test for differences between the IRRs for males versus females across the five outcomes, i.e. suicidal behaviours, substance misuse, unintentional injuries, transport accidents and criminality, yielded the following p-values: 0.81, 0.08, 0.30, 1.00 and 0.26, respectively.
